# Renal dysfunction in symptomatic Waldenström macroglobulinaemia: A nationwide Italian multicentre study

**DOI:** 10.1111/bjh.70424

**Published:** 2026-03-08

**Authors:** Nicolò Danesin, Francesco Autore, Anna Maria Frustaci, Gianmarco Favrin, Emanuele Cencini, Alessandro Noto, Irene Dogliotti, Jacopo Olivieri, Marcello Riva, Isacco Ferrarini, Anna Maria Barbui, Sara Steffanoni, Dario Marino, Benedetta Puccini, Piero Maria Stefani, Rita Rizzi, Michele Merli, Angela Ferrari, Stefano Luminari, Carlo Visco, Livio Trentin, Andrea Visentin, Annarita Conconi, Simone Ferrero, Marzia Varettoni, Alessandra Tedeschi, Luca Laurenti, Francesco Piazza

**Affiliations:** ^1^ Department of Medicine University of Padova, and Hematology Unit Azienda Ospedale Università Padova Padova Italy; ^2^ Policlinico Universitario A. Gemelli IRCCS Foundation Rome Italy; ^3^ Niguarda Cancer Center, ASST Grande Ospedale Metropolitano Niguarda Milan Italy; ^4^ Division of Hematology Fondazione IRCSS San Matteo Pavia Italy; ^5^ A.O.U. Senese and University of Siena Siena Italy; ^6^ Hematology, Fondazione IRCCS Ca' Granda, Ospedale Maggiore Policlinico Milan Italy; ^7^ Department of Molecular Biotechnologies and Health Sciences University of Torino Turin Italy; ^8^ Azienda Sanitaria Universitaria Integrata di Udine Udine Italy; ^9^ Azienda ULSS 8 Berica, Ospedale S. Bortolo Vicenza Italy; ^10^ Department of Engineering for Innovation Medicine, Section of Hematology University of Verona Verona Italy; ^11^ Azienda Ospedaliera Papa Giovanni XXIII Bergamo Italy; ^12^ U.O.C. Ematologia, Ospedale Valduce Como Italy; ^13^ Oncology 1 Unit, Veneto Institute of Oncology IOV‐IRCCS Padua Italy; ^14^ Department of Hematology AOU Careggi and University of Florence Italy; ^15^ Azienda ULSS 2 Marca Trevigiana, Ospedale Ca Foncello Treviso Italy; ^16^ Hematology and Transplant Unit, Department of Precision and Regenerative Medicine and Ionian Area University of Bari “Aldo Moro” Bari Italy; ^17^ University of Modena and Reggio Emilia Reggio Emilia Italy; ^18^ Division of Hematology Ospedale degli Infermi Biella Italy; ^19^ Hematology Division A.O.U. Città della Salute e della Scienza di Torino Torino Italy

**Keywords:** B‐cell lymphomas, IgM MGUS, monoclonal gammopathy, non‐Hodgkin lymphoma, plasma cell dyscrasia, renal insufficiency, Waldenström macroglobulinaemia

## Abstract

The prognostic significance of impaired renal function in Waldenström macroglobulinaemia (WM) remains poorly defined. We conducted a nationwide multicentre study to evaluate its clinical characteristics and prognostic impact in symptomatic patients. We analysed 402 symptomatic WM patients, stratified according to renal function at diagnosis (creatinine clearance <60 mL/min/1.73 m^2^). Renal dysfunction was identified in 119 patients (29.6%). Renal biopsy was performed in 33 cases. Patients with reduced renal function were older (median age 76 vs. 67 years, *p* < 0.0001), had lower haemoglobin levels (10.8 vs. 11.8 g/dL, *p* = 0.008) and higher 24‐h proteinuria (0.29 vs. 0.20 g, *p* = 0.04). Comorbidities of renal interest were similarly distributed between groups. Renal dysfunction was associated with inferior median overall survival (139 vs. 203 months, *p* < 0.001) and progression‐free survival (PFS) (80 vs. 106 months, *p* = 0.002). Among patients aged <70 years, renal dysfunction remained associated with shorter PFS (77 vs. 121 months, *p* = 0.005). Importantly, worsening renal function did not correlate with increasing age. In this setting, first‐line chemoimmunotherapy was associated with improved median PFS. Thus, renal dysfunction at diagnosis may represent an underrecognized, independent adverse prognostic factor in symptomatic WM.

## Graphical abstract

Renal dysfunction represents a possible underrecognized complication of symptomatic WM; this condition correlates with adverse global and disease‐specific clinical outcomes.

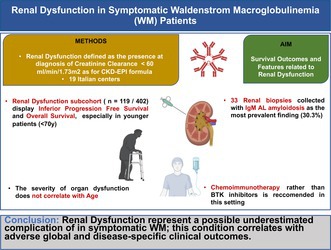

## INTRODUCTION

Renal impairment in Waldenström macroglobulinaemia (WM) may be related to WM lymphoplasmacytic cell organ infiltration or to monoclonal Immunoglobulin M (IgM)‐mediated kidney‐specific pathogenetic effects.^1^, ^2^, ^3^ Given that the median age of incidence of WM is around 68 years,^4^ besides direct disease‐related damage, renal injury may also be caused by WM‐unrelated comorbidities, such as diabetes, hypertension, other cancers, inflammatory and autoimmune disorders and medications. Identifying renal involvement caused by WM is important since it can prompt the initiation of therapy. Conversely, the presence of reduced renal function can have an impact on disease progression, treatment effectiveness and safety, representing a potential adverse prognostic condition.

Data from the literature reports a 15‐year cumulative incidence of renal involvement of 5.1% in WM, significantly lower than that described in multiple myeloma (MM), which is around 30%–40%.^5^, ^6^ In MM, cast nephropathy is the most frequent cause of kidney damage, whereas in WM, renal injury is more heterogeneous. The most frequent causes include Amyloid Light Chain (AL) or Amyloid Heavy chain (AH) amyloidosis, cryoglobulinaemic and non‐cryoglobulinaemic glomerulopathy and parenchymal tumour cell infiltration.^7^, ^8^ Additionally, rarer aetiologies such as thrombotic microangiopathy, membranous glomerulopathy and minimal change disease may also be associated with WM and other lymphoid tumours.^6^, ^9^

Determining whether renal insufficiency is WM‐related or unrelated is often challenging, necessitating a comprehensive multidisciplinary evaluation. Renal biopsy remains the gold standard for identifying the cause of renal injury; however, complications such as haemorrhage may occur, whose risk may be increased particularly in elderly patients or those on anticoagulant therapy. As a result, some patients who have a clear clinical indication for biopsy are not biopsied, leaving uncertainty regarding the origin of renal impairment.

Few studies have investigated renal involvement in WM. In a large retrospective study of 1391 WM patients, 52 cases with renal dysfunction (defined as estimated Glomerular Filtration Rate (eGFR) <60 mL/min/1.73 m^2^ or abnormal 24‐h proteinuria) who underwent kidney biopsy were identified, with 44 confirming WM‐related kidney damage. The most common histopathological alterations observed were AL amyloidosis (25%), cryoglobulinaemic glomerulonephritis (GN) (23%) and tubulointerstitial infiltrate/damage (18%). The median overall survival (OS) for WM patients with renal involvement was lower (11.5 vs. 16 years, log‐rank *p* = 0.03). The most used first‐line regimens in 28/44 WM patients with renal dysfunction were proteasome inhibitor + rituximab (50%), alkylating agent + rituximab (25%) and other regimens (25%). Disease response correlated with organ response, as measured by eGFR.^7^ Another study analysing 42 cases with available renal biopsies demonstrated a WM/LPL‐related renal involvement but did not find a correlation between haematological response and renal response.^10^ Other smaller and less selective cohorts have been described.^11^, ^12^

Prospective studies on immunochemotherapy regimens or new‐generation drugs generally did not include sufficient patients with moderate‐to‐severe renal dysfunction or direct organ involvement. For example, the European Consortium on WM trial comparing Bortezomib‐Dexamethasone Rituximab Cyclophosphamide (B‐DRC) versus rituximab–cyclophosphamide–dexamethasone (DRC) included patients with WM‐related nephropathy, but required a creatinine level of <2 mg/dL before enrolment.^13^ Similarly, the phase 2 study on BDR (bortezomib, dexamethasone, rituximab) as first‐line therapy in WM required a creatinine clearance >30 mL/min/1.73 m^2^.^14^ Additionally, most prospective trials lack dedicated analyses of outcomes for patients with renal impairment. Trials leading to the approval of Bruton's tyrosine kinase inhibitors (BTKis) for WM also required adequate organ function for enrolment, although the iNNOVATE study set the eGFR threshold at >30 mL/min/1.73 m^2^.^15^, ^16^ Real‐world cohorts studying commonly used regimens like rituximab–bendamustine often excluded patients with creatinine levels >2 mg/dL, although a recent real‐life Italian study included WM patients with lower Creatinine Clearance.^17^, ^18^ The last consensus panel of the 11th International Workshop on Waldenstrom Macroglobulinemia suggested the superiority of bortezomib‐based regimens in WM patients with renal dysfunction, drawing from insights gained in MM studies.^19^

Overall, there is a paucity of data regarding outcomes, efficacy and safety of therapy in WM patients with reduced renal function. To address this issue, we conducted a nationwide multicentre retrospective study analysing the clinical–biological features and survival outcomes of a large cohort of WM patients. We compared those with renal impairment to those without and performed a sub‐analysis on cases with biopsy‐proven organ involvement.

## METHODS

### Cohort characteristics

This retrospective, multicentre, observational study was conducted following approval from the Ethics Committee of Azienda Ospedale Università Padova (authorization #5922/AO/24, ID22770). A written informed consent was given to all the participants. The study included 471 symptomatic WM patients, whose data were collected independently from 18 Italian centres affiliated with the Fondazione Italiana Linfomi.

Patients were eligible if they had a confirmed diagnosis of WM between 1 January 2000 and 1 January 2023, according to the 5th edition of WHO classification and ICC 2022 criteria, were aged ≥18 years, had received at least one line of therapy for symptomatic or active disease and had complete data available for analysis.

Renal dysfunction was defined as a creatinine clearance <60 mL/min/1.73 m^2^ at diagnosis, calculated with the CKD‐EPI (Chronic Kidney Disease—Epidemiology Collaboration) creatinine equation.^20^ To assess the impact of organ impairment, renal dysfunction was stratified into three categories: mild (45–60 mL/min/1.73 m^2^), moderate (44–30 mL/min/1.73 m^2^) and severe (<30 mL/min/1.73 m^2^).

Response to first‐line therapy, including in patients diagnosed before 2013, was assessed according to the 6th International Workshop consensus criteria for WM.^21^ The International Prognostic Scoring System for WM (IPSSWM) was calculated in accordance with published consensus guidelines.^21^, ^22^ A predefined subanalysis distinguished patients with biopsy‐confirmed WM‐related renal involvement. Renal biopsies were performed at the discretion of the treating centre at any time during follow‐up and were not centrally reviewed. Based on the median age at diagnosis, patients were classified as younger (<70 years) or older (≥70 years).^4^ The Cumulative Illness Rating Scale (CIRS) was calculated according to standard definitions.^23^ Detection of the *MYD88* ^L265P^ mutation on bone marrow aspirates was performed in most cases using highly sensitive allele‐specific polymerase chain reaction (PCR) (AS‐PCR) or digital droplet PCR (ddPCR). *CXCR4* mutations were assessed on bone marrow aspirate using two AS‐PCR assays, targeting the c.1013C > G mutation and the c.1013C > A mutation, respectively; sequencing of exon 2 was additionally performed to identify other non‐sense or frameshift variants.

Renal comorbidities were defined as conditions potentially contributing to renal damage, including hypertension, diabetes mellitus, benign urological conditions and urothelial malignancies. Treatment modification was defined as a reduction in the number of treatment cycles and/or drug dosage.

### Statistical analysis

Continuous variables were summarized using the median and interquartile range (IQR), and categorical variables as frequencies and percentages. Comparisons between groups were performed using the Mann–Whitney *U*‐test for continuous variables and the chi‐squared test or Fisher's exact test, as appropriate, for categorical variables.

Survival end‐points included OS, progression‐free survival (PFS) and time to next treatment (TTNT). OS was defined as the time from diagnosis to death from any cause; PFS as the time from diagnosis to disease progression or death from any cause; and TTNT as the time from initiation of first‐line therapy to commencement of second‐line treatment.

Overall response rate (ORR) comprised complete response (CR), very good partial response (VGPR), partial response (PR) and minor response (MR). Major response rate (MRR) was defined as ORR excluding minor response. Cumulative incidence of disease progression was calculated from the time of diagnosis.

Within the renal dysfunction subcohort, the impact of variables on OS, PFS and TTNT was assessed using the Cox proportional hazard regression in univariable and multivariable analyses. Only the variables with significantly different distribution between the two subcohorts at baseline were included in the analysis.

Results were expressed as hazard ratios (HRs) with 95% confidence intervals (CIs). Patients with missing data were excluded from the analyses.

Survival curves were estimated using the Kaplan–Meier method and compared using the log‐rank test. A two‐sided *p* < 0.05 was considered statistically significant. Statistical analyses were performed using RStudio (version 2023.12.1 + 402).

## RESULTS

### Clinical features

After exclusion of 69 patients due to incomplete data, 402 patients were included in the final analysis of whom 119 (29.6%) had renal dysfunction at diagnosis (Table 1). Patients with renal dysfunction were older (median age 76 vs. 67 years, *p* < 0.0001), had lower median haemoglobin levels (10.8 vs. 11.8 g/dL, *p* = 0.008), higher median 24‐h proteinuria (0.29 vs. 0.20 g, *p* = 0.04) and lower prevalence of kappa‐restricted paraprotein (77% vs. 90%, *p* = 0.002) compared with those with preserved renal function. In contrast, indicators of disease burden—including serum monoclonal IgM levels, bone marrow infiltration, lactate dehydrogenase (LDH) and β2‐microglobulin levels—were comparable between groups, as was the prevalence of *MYD88* ^L265P^ and *CXCR4* mutations. A trend towards a higher proportion of intermediate‐risk IPSSWM patients was observed in the renal dysfunction subgroup, largely attributable to older age. CIRS scores were similar between groups. Notably, comorbidities potentially affecting renal function—such as hypertension, diabetes mellitus and pre‐existing chronic kidney disease—were equally distributed across subgroups.

**TABLE 1:** Baseline clinical and biological characteristics of sWM patients according to renal function status.

	Renal dysfunction (*n* = 119)	Normal renal function (*n* = 283)	*p*‐value
Median age, years, median (IQR)	76 (69–80)	67 (58–74)	**<0.0001**
Male, *n* (%)	75/119 (63.0)	181/283 (64)	0.86
CIRS > 6, *n* (%)	22/119 (17)	57/283 (21)	0.70
Renal comorbidites^a^, *n* (%)	8/119 (5)	11/283 (5)	0.22
Urotelial malignancies, *n* (%)	2/116 (2)	6/279 (2)	0.99
24‐h proteinuria, g/24 h, median (IQR)	0.29 (0.13–1.77)	0,20 (0,12‐0,41)	**0.04**
24‐h proteinuria >5 g/24 h, *n* (%)	19/66 (29)	20/101 (20)	0.18
Renal biopsies, *n* (%)	21/119 (17)	12/283 (4)	**<0.0001**
Hb levels, g/dL, median (IQR)	10.8 (9.5–12.4)	11.8 (10.0–13.2)	**0.008**
PLT count, ×10^9^/L, median (IQR)	247.0 (187.5–311.5)	225.0 (160.0–293.0)	0.15
IgM levels, mg/dL, median (IQR)	2340.0 (1188,0–4025.0)	2204,0 (1000.0–4020.0)	0.72
IgM kappa, *n* (%)	92/119 (77)	255/283 (90)	**0.002**
Bone marrow infiltration, %, median (IQR)	60 (30–80)	55 (30–75)	0.22
LDH > UNL, *n* (%)	25/109 (23)	63/268 (24)	0.91
Albumin <3.5, g/L, *n* (%)	41/99 (41)	84/239 (35)	0.33
β2‐microglobulin ≥3, mg/L, *n* (%)	72/95 (76)	173/242 (71)	0.50
*MYD88* ^L265P^, *n* (%)	34/38 (89)	172/184 (93)	0.49
*CXCR4* mutations, *n* (%)	4/19 (21)	15/53 (28)	0.54
IPSSWM, *n* (%)			
Low	24/108 (22)	66/259 (26)	0.20
Intermediate	53/108 (49)	101/259 (39)	
High	31/108 (28)	92/259 (36)	
r‐IPSSWM, *n* (%)			
Very Low	25/110 (23)	60/254 (24)	0.36
Low	53/110 (48)	103/254 (41)	
Intermediate	19/110 (17)	52/254 (20)	
High	9/110 (8)	31/254 (12)	
Very high	4/110 (4)	8/254 (3)	

*Note*: Significant (*p* <0.05) comparison are in bold.

Abbreviations: IQR = interquartile range; CIRS = Cumulative IIlness Rating Scale (CIRS), Hb = haemoglobin, PLTs = platelets, LDH = lactate dehydrogenase, UNL = upper normal level, IPSSWM = International Prognostic Scoring System on Waldenström macroglobulinaemia.

^a^ Hypertension, diabetes, pre‐existing chronic renal insufficiency.

### Histological patterns

Of 33 renal biopsies, 21 were performed at diagnosis and 12 subsequently in patients with renal dysfunction considered to be of unexplained origin.

**FIGURE 1:**
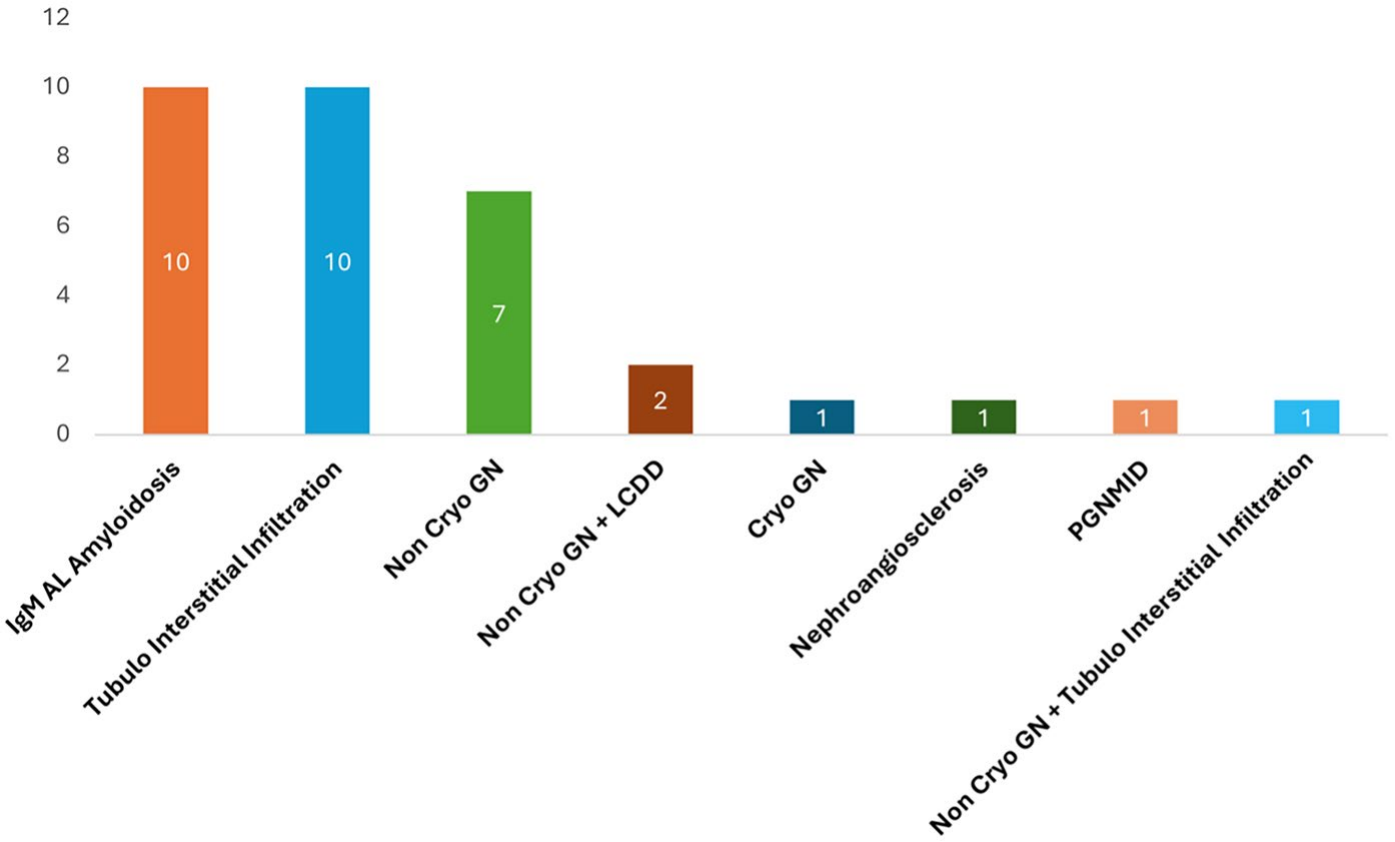
Distribution of renal histopathological patterns in biopsied symptomatic waldenstrom macroglobulinemia (sWM) patients (*n* = 33).

The observed histopathological patterns included IgM AL amyloidosis (30.3%), tubulo‐interstitial infiltration (30.3%), non‐cryoglobulinaemic GN (21.2%), combined non‐cryoglobulinaemic GN and light chain deposition disease (6.2%), cryoglobulinaemic GN (3.0%), combined non‐cryoglobulinaemic GN and tubulo‐interstitial infiltration (3.0%), proliferative GN with monoclonal immunoglobulin deposits (PGNMID) (3.0%) and nephroangiosclerosis (3.0%) (Figure 1).

Overall, glomerular involvement—including amyloidosis, GN, PGNMID and light chain deposition disease—represented the predominant pattern type of renal injury, occurring in 21 of 33 cases (63.6%).

### Management

Chemoimmunotherapy (CIT) was the most frequently used first‐line treatment in both patients with renal dysfunction and those with preserved renal function (61% vs. 68%, not significant). CIT regimens included bendamustine–rituximab (BR, the most used), DRC and rituximab–chlorambucil. No significant differences were observed between groups in terms of reductions in the number of therapy cycles or dosage adjustments. The incidence and spectrum of haematological and non‐haematological adverse events were comparable. (Table 2).

**TABLE 2:** First‐line treatment regimens, treatment modifications, toxicity and efficacy in sWM patients according to renal function status.

	Renal dysfunction (*n* = 119)	Normal renal function (*N* = 283)	*p*‐value
CIT, *n* (%)	74/119 (62)	191/283 (68)	n.s.
BR	48/119 (40)	126/283 (45)	n.s.
RCD	26/119 (22)	63/282 (22)	n.s.
R‐Chlorambucil	—	2/282 (1)	n.s.
Bortezomib‐based regimens^a^, *n* (%)	5/119 (4)	5/283(2)	n.s.
Other^b^, *n* (%)	40/119 (34)	87/279 (32)	n.s.
Treatment modification, *n* (%)	33/119 (28)	59/283 (21)	n.s.
Dose reduction, *n* (%)	20/119 (17)	31/272 (11)	n.s.
Cycle reduction, *n* (%)	21/119 (18)	41/280 (15)	n.s.
Haem toxicity G3, *n* (%)	11/119 (9)	14/283 (5)	n.s.
No haem toxicity G3, *n* (%)	9/119 (8)	16/283 (6)	n.s.
MRR, *n* (%)	90/119 (75)	207/270 (77)	n.s.
ORR, *n* (%)	101/116 (84)	234/270 (87)	n.s.
CR	15/119 (12)	44/270 (16)	n.s.
VGPR	10/119 (9)	45/270 (17)	**0.03**
PR	65/119 (54)	118/270 (44)	n.s.
MR	11/119 (9)	27/270 (10)	n.s.
SD	10/119 (9)	20/270 (7)	n.s.
PD	8/119 (6)	16/270 (6)	n.s.
Death events, *n* (%)	32/119 (27)	53/279 (19)	**0.08**
Progression events, *n* (%)	47/119 (40)	113/283 (40)	n.s.

*Note*: Significant (*p* <0.05) comparison are in bold.

Abbreviations: BR, bendamustine–rituximab; CIT, chemoimmunotherapy; CR, complete response; G3, grade 3; MR, minor response; MRR, major response rate; n.s., not significant; ORR, overall response rate; PD, progressive disease; PR, partial response; RCD, rituximab–cyclophosphamide–dexamethasone; SD, stable disease; VGPR, very good partial response.

^a^ Bortezomib or bortezomib–rituximab–dexamethasone.

^b^ Ibrutinib, zanubrutinib, rituximab monotherapy, chlorambucil monotherapy.

ORR and MRR were similar between the two subgroups (Table 2). Similarly, no significant differences in ORR and MRR were observed among patients treated with BR, according to renal function status (Table S1). Among patients with renal dysfunction, those receiving CIT achieved higher ORRs compared with alternative first‐line regimens (89% vs. 70%, *p* = 0.009) (Table S2).

Notably, among patients with decreased renal function, those who underwent renal biopsy at diagnosis more frequently received first‐line bortezomib‐based regimens (19% vs. 1%, *p* < 0.001) and Rituximab Cyclophosphamide Dexamethasone (RCD) (38% vs. 18%, *p* = 0.05) compared with non‐biopsied patients. However, ORR and MRR were similar between these subgroups (Table S3). In the biopsied cohort, first‐line treatments included BR (9/33), DRC (11/33), bortezomib‐based regimens (7/33) and 6/33 other therapies.

### Survival analysis and survival outcomes

Median follow‐up was 95.4 months (IQR 86.8–108.6) for the entire cohort, 78.9 months (59.3–103.3) for patients with renal dysfunction and 106.2 months (92.1–124.9) for those with preserved renal function.

**FIGURE 2:**
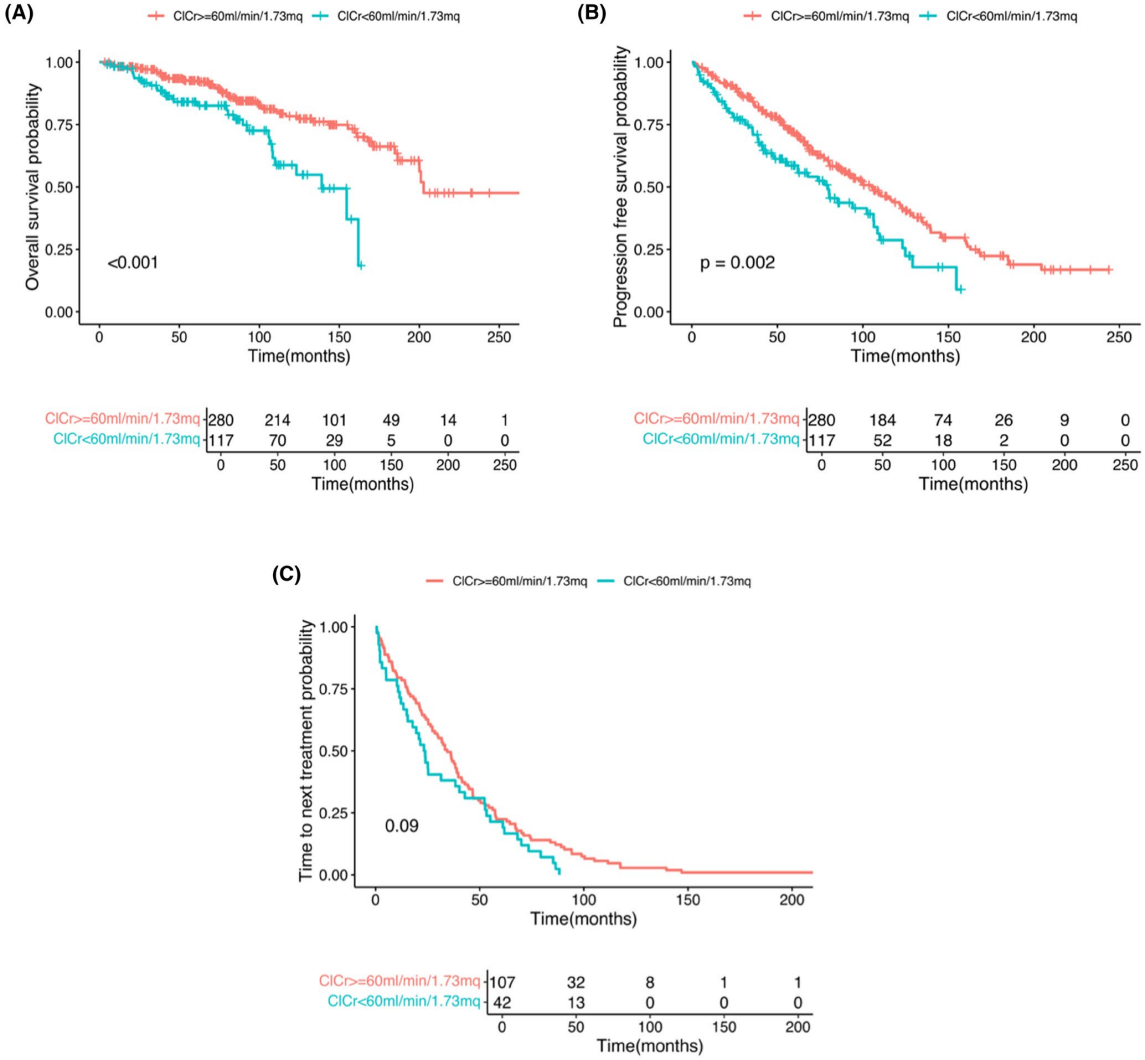
Overall survival (A), progression‐free survival (B) and time to next treatment (C) in sWM patients according to renal function status.

Patients with renal dysfunction experienced significantly inferior median OS (139 vs. 203 months, *p* < 0.001; HR 2.33, 95% CI 1.47–3.72) and median PFS (80 vs. 106 months, *p* = 0.002; HR 1.61, 95% CI 1.19–2.19). A trend towards shorter median TTNT was also observed (24 vs. 34 months, *p* = 0.09; HR 1.41, 95% CI 0.97–2.03) (Figure 2, Table 3).

**TABLE 3:** Overall survival, progression‐free survival and time to next treatment in sWM patients according to renal function status.

	Renal dysfunction	No renal dysfunction	HR (IC 95%)	*p*‐value
Median OS	139	203	2.33 (1.47–3.72)	**<0.001**
Median PFS	80	106	1.61 (1.19–2.19)	**0.002**
Median TTNT	24	34	1.41 (0.97–2.03)	0.09

*Note*: Significant (*p* <0.05) comparison are in bold.

Abbreviations: HR, hazard ratio; IC, interval confidence; OS, overall survival; PFS, progression free survival; TTNT, time to next treatment.

The cumulative incidence of disease progression was higher in patients with renal dysfunction at both 100 months (38% vs. 28%) and 200 months (58% vs. 52%; *p* = 0.05) (Figure S1).

In univariable Cox regression within the renal dysfunction subgroup, advanced age was associated with inferior PFS, OS and TTNT.

**FIGURE 3:**
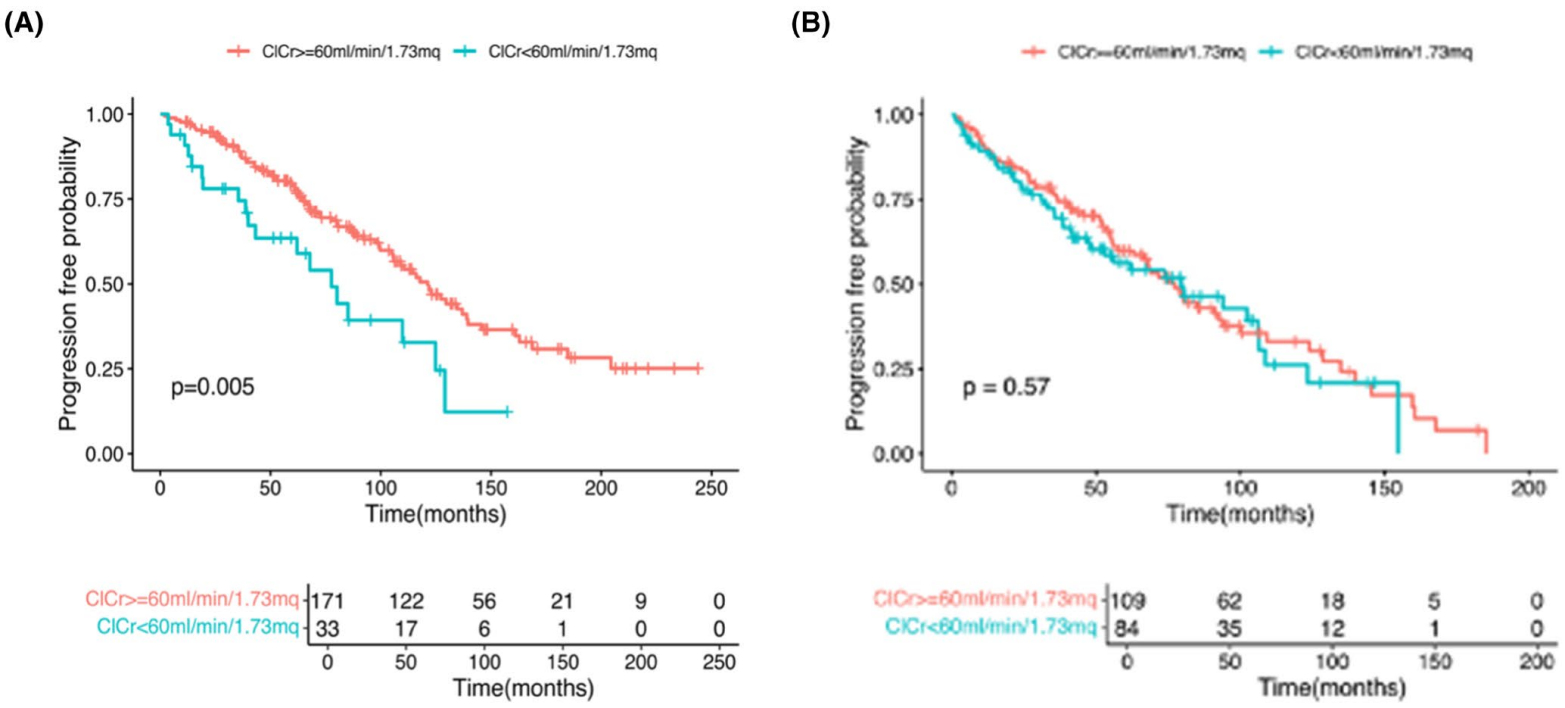
Progression‐free survival in sWM patients aged < 70 years (A) and ≥ 70 years (B) stratified by renal function status.

To further explore the impact of age, patients were stratified into those aged <70 and ≥ 70 years. Renal impairment was less prevalent in younger patients (15% vs. 43%, *p* < 0.0001); however, younger patients with renal dysfunction had significantly shorter PFS compared to those with preserved renal function (77 vs. 121 months, *p* = 0.005). No difference in PFS was observed among older patients according to renal dysfunction (77 vs. 79 months; *p* = 0.57) (Figure 3). In younger patients, OS and TTNT did not differ by renal function (Figure S2), whereas in older patients, renal dysfunction was associated with inferior median OS (108 vs. 161 months, *p* = 0.04; HR 1.79, 95% CI 1.03–3.15) (Figure S3).

Among younger patients with renal dysfunction, those who underwent a renal biopsy had inferior PFS on univariable analysis (HR 5.51, *p* = 0.02); no other clinical or pathological variables were associated with outcome in this subgroup.

Consistent with prior observations, older patients with preserved renal function had inferior OS (not reached vs. 155 months, *p* < 0.0001), PFS (77 vs. 121 months, *p* < 0.0001) and TTNT (25 vs. 41 months, *p* = 0.004) compared with younger counterparts (Figures S4 and S5).^4^ Severity of renal dysfunction at diagnosis, stratified as low (creatinine clearance 60–45 mL/min/1.73 m^2^), intermediate (44–30 mL/min/1.73 m^2^) and severe (<30 mL/min/1.73 m^2^), was not associated with higher age (Table S4) nor with differences in OS, PFS or TTNT (Figure S6).

Among patients with histologically confirmed WM‐related kidney damage, no association was observed between histopathological patterns and outcomes. In particular, patients with amyloidosis had outcomes comparable to those with other renal lesions. OS, TTNT and PFS outcomes were also similar between biopsied and non‐biopsied patients in the entire cohort (Figure S7). Additional details regarding this sub cohort are extensively reported in Table S5.

Notably, among patients treated with BR, those with renal dysfunction demonstrated superior median PFS in both univariable and multivariable analyses (Table 4, Figure S8). Consistent with this, CIT was associated with higher ORR in patients with impaired renal function (89% vs. 70%, *p* = 0.009).

**TABLE 4:** Univariate and multivariate Cox proportional hazard analyses of prognostic factors in sWM with renal dysfunction.

	Univariate OS renal dysfunction			Multivariate OS renal dysfunction		
	HR	IC 95%	*p* value	HR	IC 95%	*p* value
Age	1.15	1.08–1.23	**<0.0001**	—	—	—
BR	—	—	—	—	—	—
Hb	—	—	—	—	—	—

	Univariate PFS renal dysfunction			Multivariate PFS renal dysfunction		
	HR	IC 95%	*p* value	HR	IC 95%	*p* value
Age	1.03	1.00–1.06	0.06	—	—	—
BR	0.4	0.26–0.72	**0.001**	0.43	0.24–0.77	**0.004**
Hb	—	—	—	—	—	—

	Univariate TTNT renal dysfunction			Multivariate TTNT renal dysfunction		
	HR	IC 95%	*p* value	HR	IC 95%	*p* value
Age	1.05	1.01–1.10	**0.02**	—	—	—
BR	—	—	—	—	—	—
Hb	—	—	—	—	—	—

*Note*: Significant (*p* <0.05) comparison are in bold.

Abbreviations: BR, bendamustine, rituximab; Hb, haemoglobin; HR, hazard ration; IC, confidence interval; OS, overall survival; PFS, progression‐free survival; TTNT, time to next treatment.

## DISCUSSION

In this study, we analysed a large, nationwide, Italian multicentre cohort of sWM patients, comparing cases with reduced to cases with preserved renal function, both with and without direct WM‐related organ involvement. Renal impairment was defined according to Kidney Disease: Improving Global Outcomes guidelines, using an eGFR of <60 mL/min/1.73 m^2^ at diagnosis; 24‐h proteinuria, serum‐free light chain and Bence Jones proteinuria were not included due to inconsistent collection across centres. The primary objective was to assess the impact of reduced renal function on disease outcomes.

Renal dysfunction at diagnosis was present in 119 of 402 patients (29%), a fraction higher than previously reported in other retrospective series.^7^ Patients with renal impairment exhibited inferior median OS and PFS. BR was the only regimen associated with significantly improved PFS, retaining significance in multivariable analysis. Although the renal dysfunction subgroup had an older median age, which in part may account for the observed higher rate of renal impairment, there was no increased prevalence of age‐related renal comorbidities, urothelial malignancies or high CIRS scores, suggesting that age alone or a superior prevalence of comorbidities could only partially explain our observation. Thus, it is possible that a fraction of patients may have had undiagnosed WM‐related renal involvement even in case of low clonal disease burden.

The higher cumulative incidence of progression in patients with renal dysfunction suggests a potentially more aggressive disease course. Cox regression showed that age impacted outcomes in univariable but not multivariable models. Notably, younger patients (<70 years) with renal impairment had significantly shorter PFS than their counterparts without organ impairment, whereas no difference was observed in older patients (≥70 years). The severity of renal dysfunction did not correlate with age or further stratify OS, PFS and TTNT, supporting the hypothesis that renal dysfunction itself is an independent adverse prognostic factor.

Renal impairment did not lead to significant therapy modifications or increased treatment‐related toxicity, and ORR and MRR were comparable between groups. Only 33 patients with unexplained renal dysfunction underwent renal biopsy, likely reflecting procedural risks, need for specialized expertise and resources for post‐procedure monitoring and clinical contraindications by physician judgement. Biopsied patients were more frequently treated with bortezomib‐based regimens or DRC, possibly reflecting physician preference for therapies effective in paraprotein‐related renal damage. Moreover, histopathological patterns did not seem to correlate with outcomes.

In conclusion, renal impairment in WM is associated with inferior PFS and OS. BR and other CIT regimens appear effective first‐line options, while bortezomib‐based therapies remain valid alternatives. Patients under 70 years with renal dysfunction are at particular risk of shorter PFS. Despite a trend towards older age in the renal dysfunction subgroup, comorbidities affecting renal function were evenly distributed, and worsening renal impairment was not age dependent. The low rate of renal biopsy may underestimate organ involvement in WM.

Our study provides novel insights into the outcome of WM patients with renal impairment, especially in the era of targeted agents, where such patients are underrepresented in clinical trials. It also suggests that direct organ damage by WM may be present in patients with unexplained renal dysfunction, for whom a renal biopsy should be considered. Limitations include the retrospective nature of the analysis, the low number of histological data and the incomplete data for 24‐h proteinuria, serum‐free light chain measurements and urinary Bence Jones protein. This latter aspect could limit the interpretation of results in case of renal amyloidosis or light chain deposition disease damage.

Prospective studies with larger cohorts of renal‐biopsied patients are warranted to further clarify the prognostic and therapeutic implications, especially with novel drugs, of renal involvement and renal dysfunction in WM.

## AUTHOR CONTRIBUTIONS

ND and FP involved in study conception, data analysis and writing. ND, FA, AMF, GF, EC, AN, ID, JO, MR, IF, AMB, SS, DM, BP, PMS, RR, MM, AF, SL, CV, LT, AV, AC, SF, MV, AT, LL and FP involved in data contribution and revision of the study.

## FUNDING INFORMATION

The authors received no funding for this study.

## CONFLICT OF INTEREST STATEMENT

The authors have no conflict of interest to disclose.

## ETHICS STATEMENT

This work has been revised and approved by the Ethical Committee of the Azienda Ospedale Università Padova.

## PATIENT CONSENT STATEMENT

Informed consent was obtained from all participants in the study. Personal data have been rendered anonymous.

## PERMISSION TO REPRODUCE MATERIAL FROM OTHER SOURCES

The authors allow to reproduce material from other sources upon reasonable request to the corresponding author.

## Supporting information


Figure S1.

## Data Availability

The data that sustain the findings of this study are available from the corresponding author (francesco.piazza@unipd.it) upon reasonable request.
