# Supplementary material for: Renal dysfunction in symptomatic Waldenström macroglobulinaemia: A nationwide Italian multicentre study
Source: Br J Haematol. 2026 Mar 8;208(6):2059–68. doi: 10.1111/bjh.70424 (PMC13267470; doi:10.1111/bjh.70424)
Supplement: Supplementary file 1 — Figure S1. [file BJH-208-2059-s001.docx]

**Figure S1.** Cumulative incidence of disease progression in sWM patients with (green) and without (red) renal dysfunction.

**
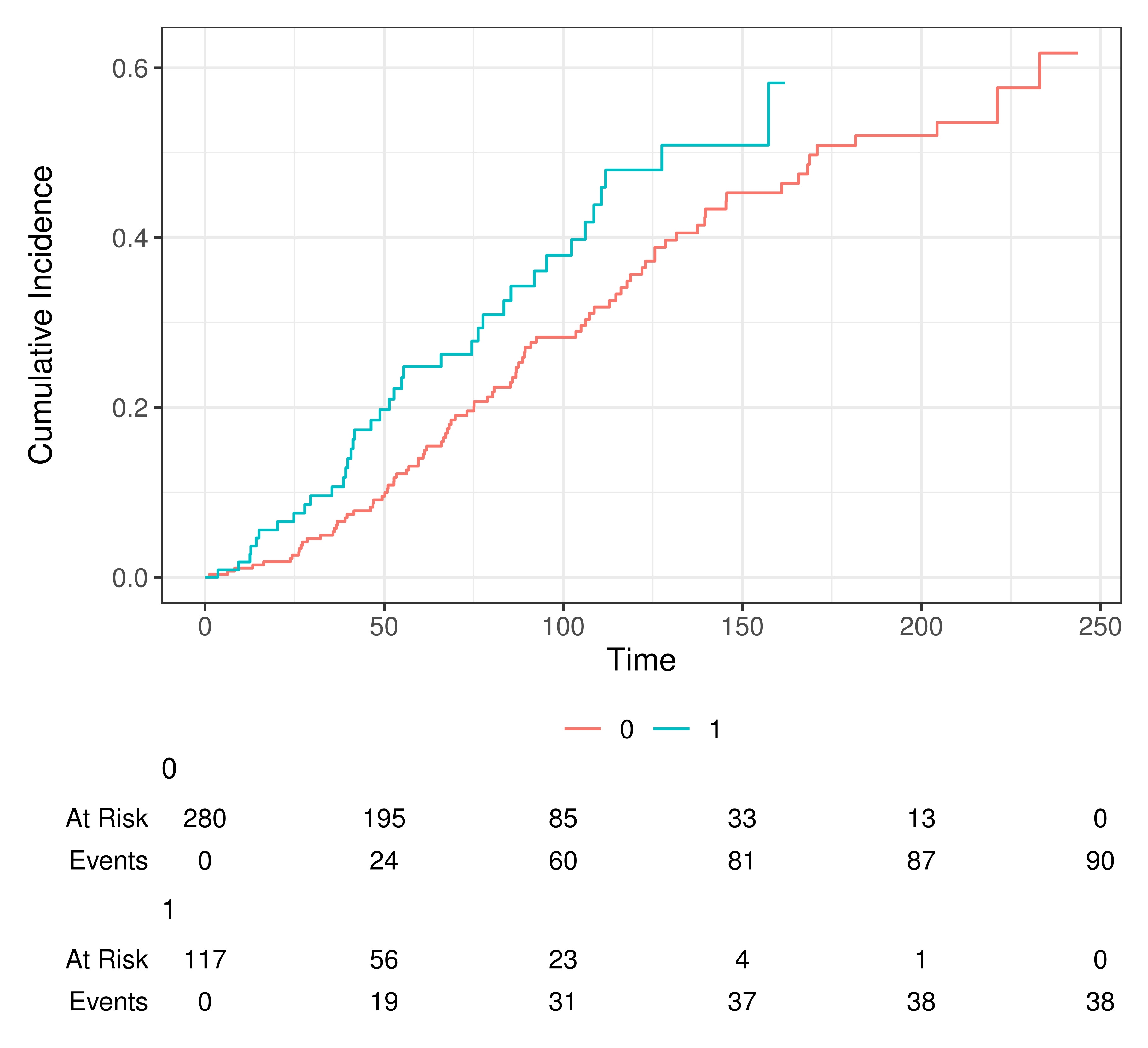
**

**Figure S2.** OS, TTNT in sWM patients aged < 70 years with (green) and without (red) renal dysfunction.

1. **
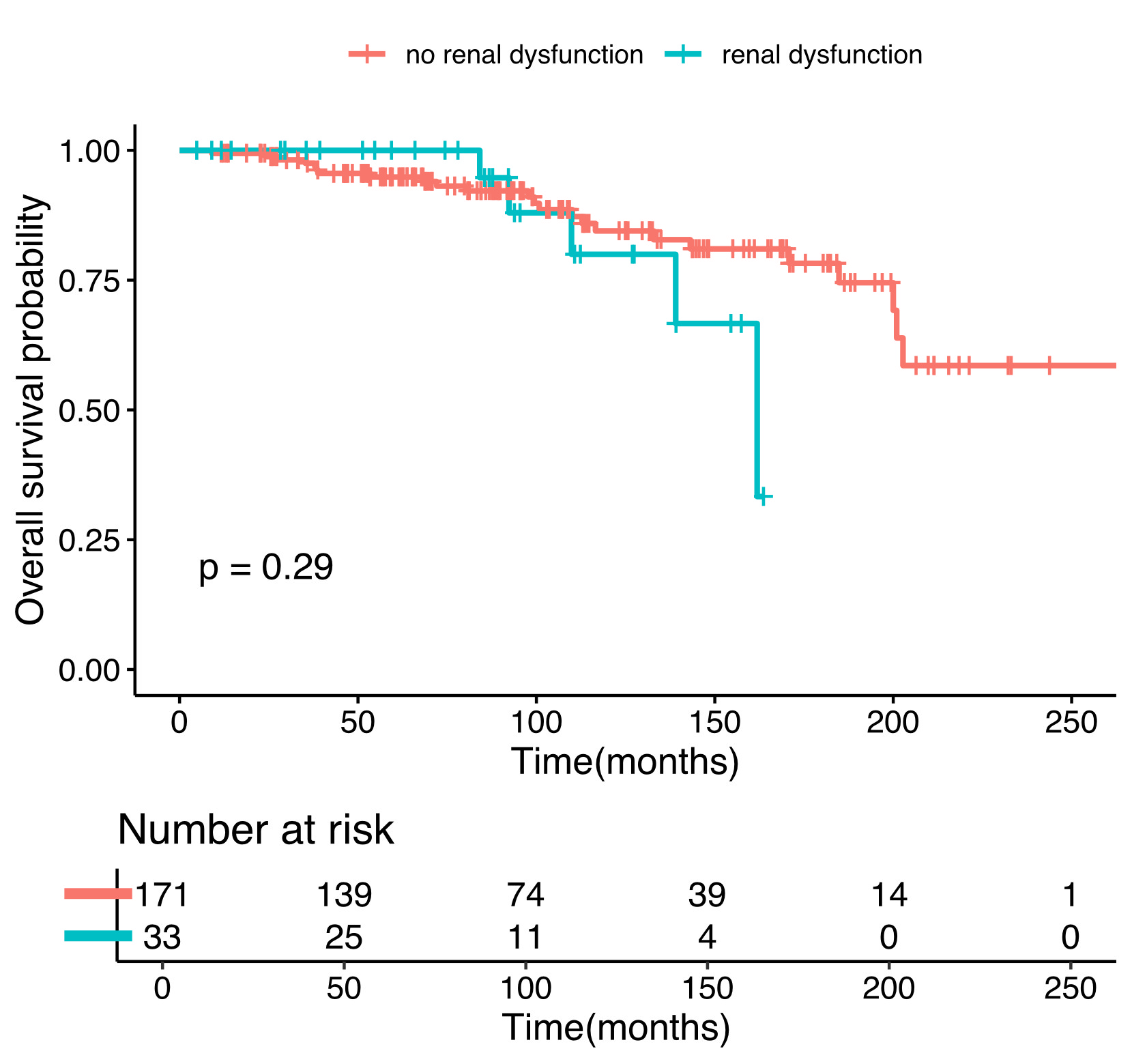
 B)
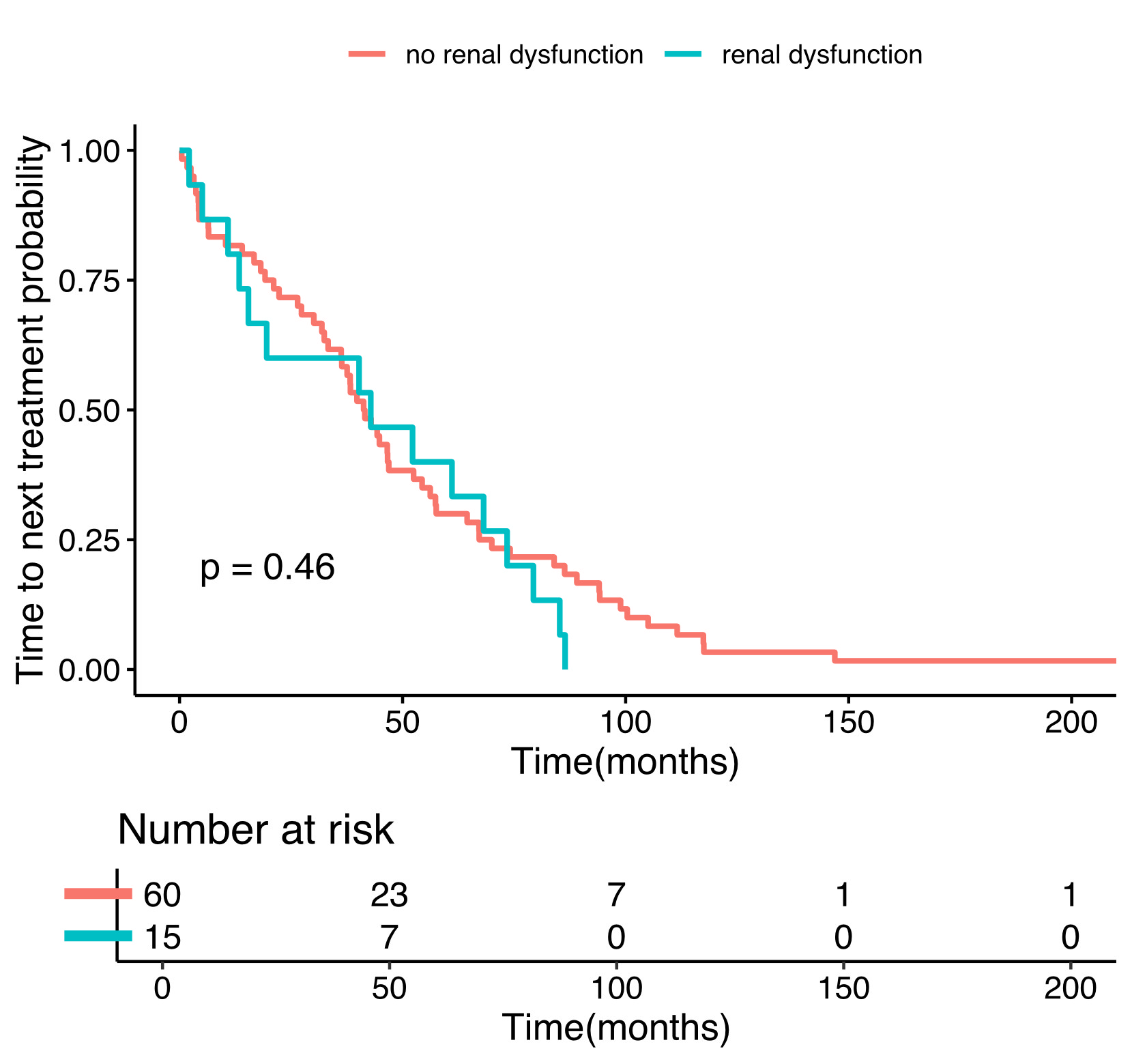
**

**Figure S3.** OS, TTNT in sWM patients aged ≥70 with (green) and without (red) renal dysfunction.

**A)
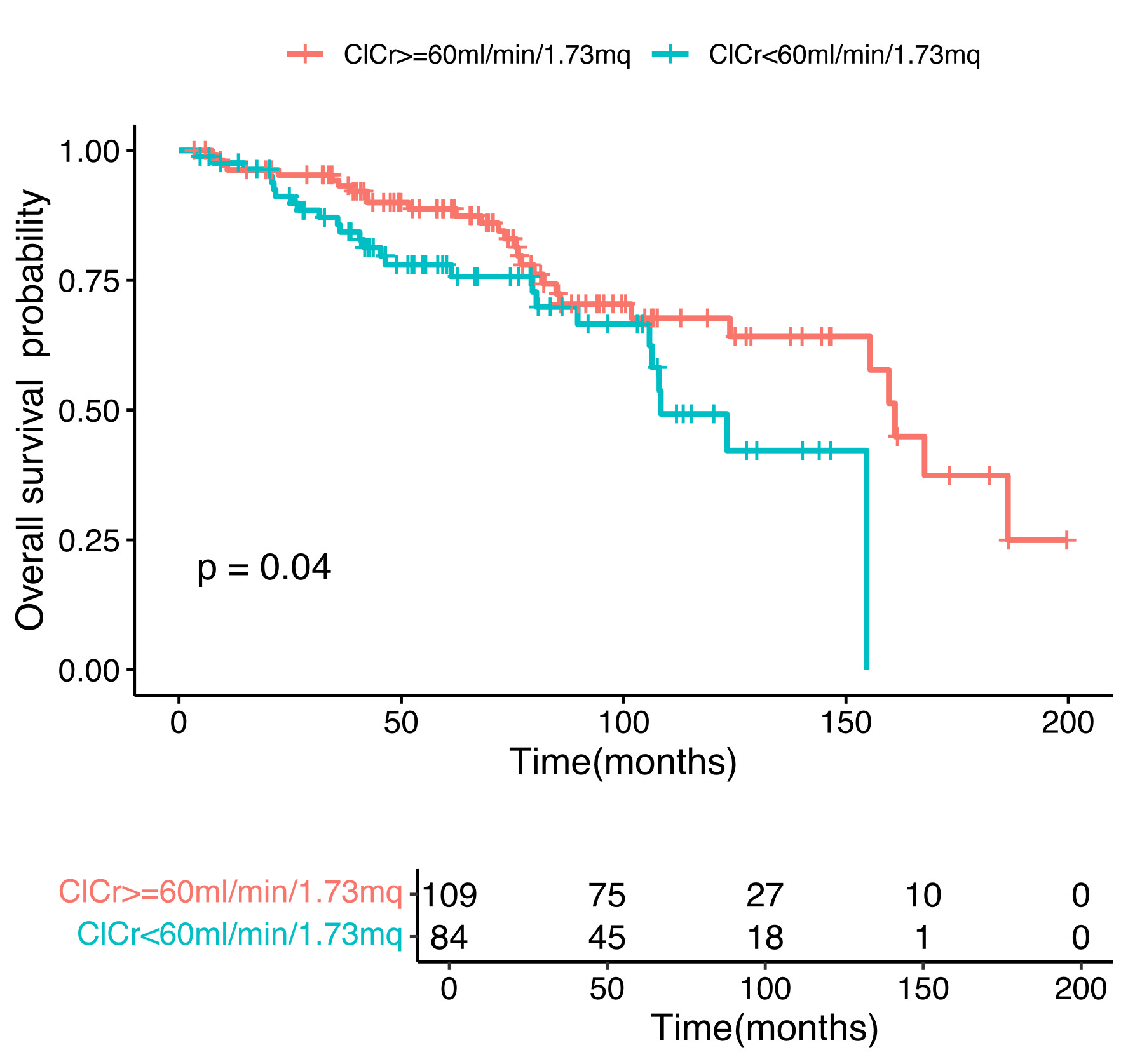
 B)
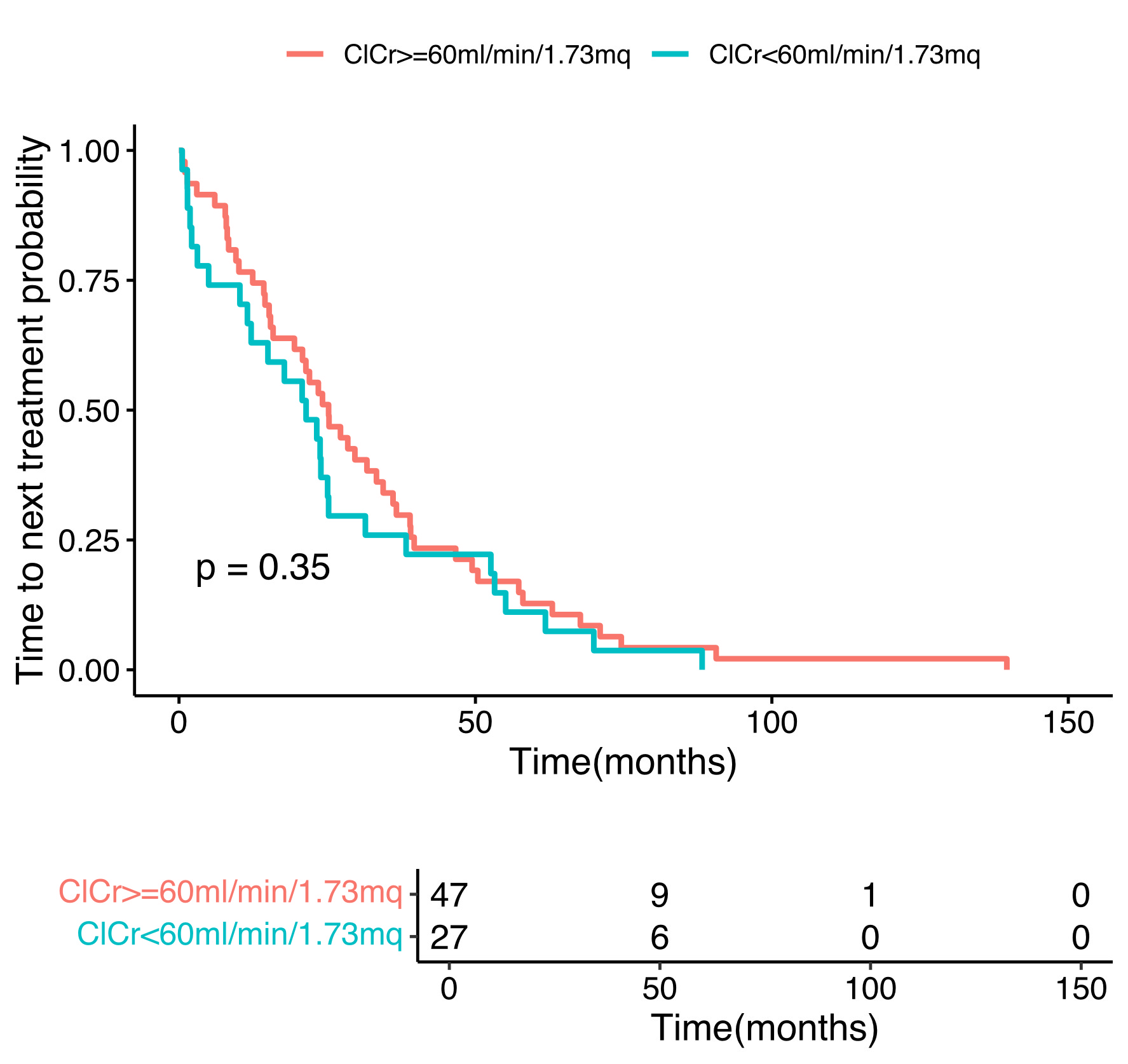
**

**Figure S4.**TTNT, OS, PFS in sWM patients with renal dysfunction aged ≥ 70 years (green) and < 70 years (red).

1.
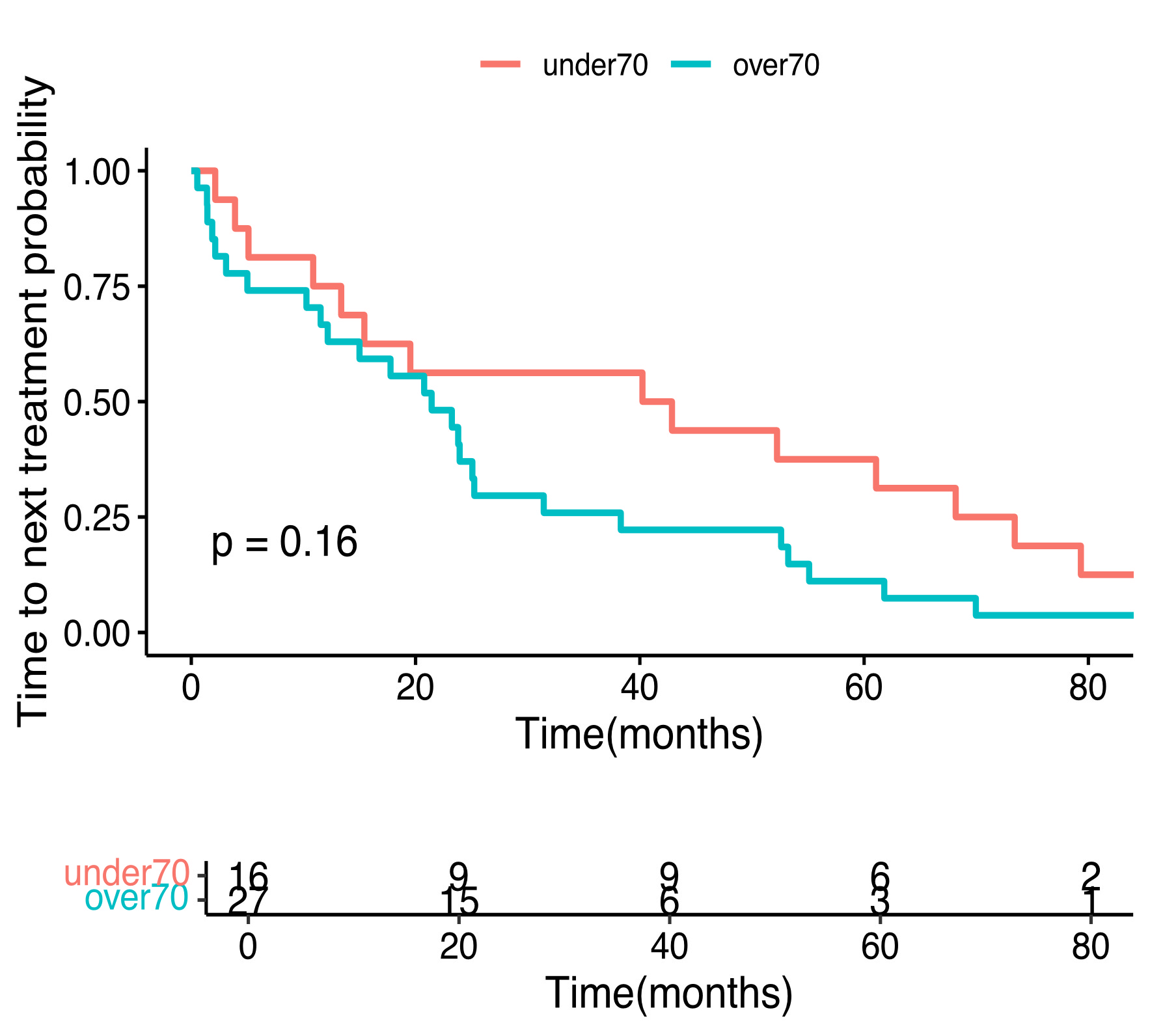
 B)
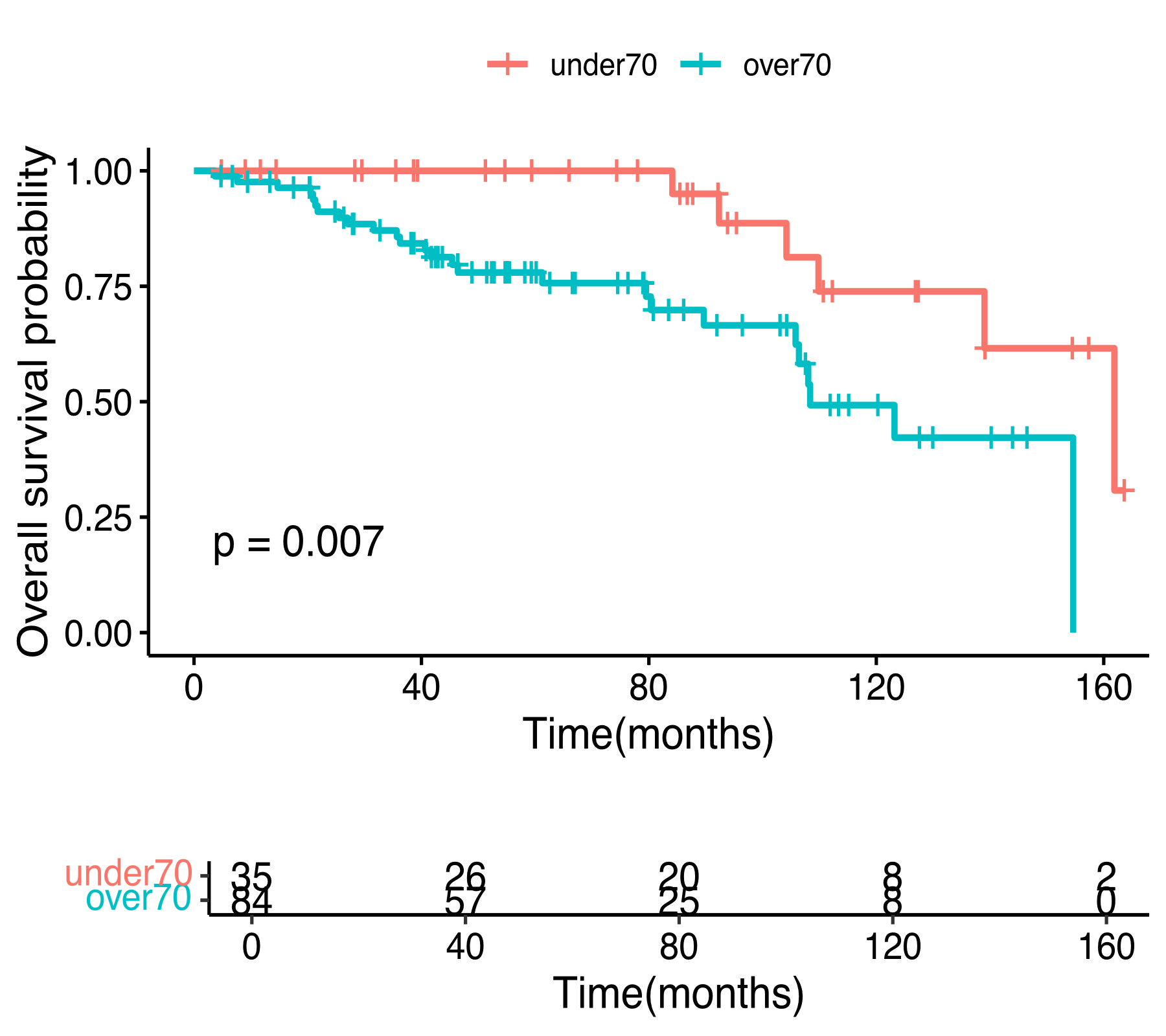

2.
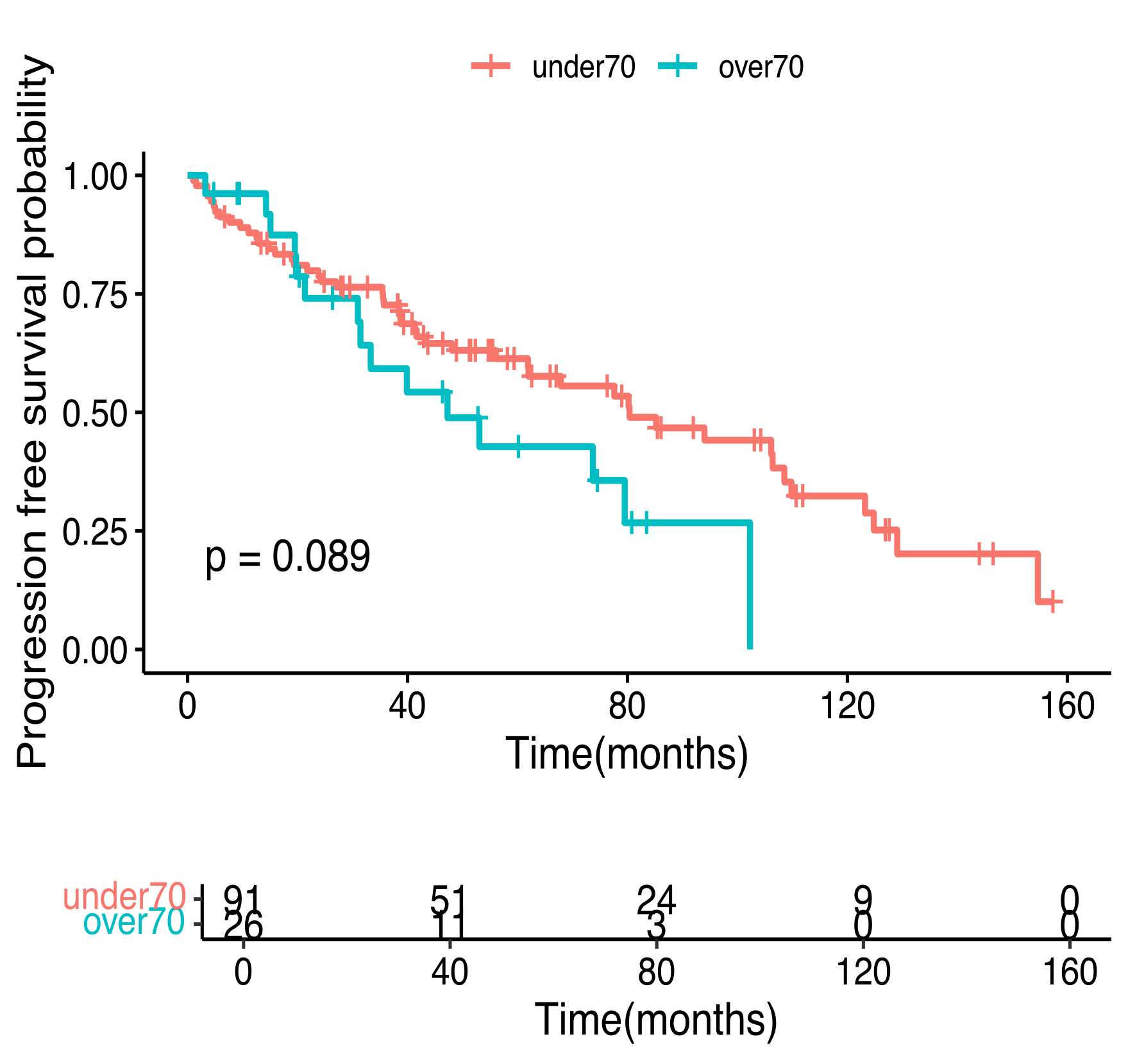


**Figure S5.** TTNT, PFS, OS in sWM with preserved renal function aged ≥ 70 years (green) and < 70 years (red).

1. **
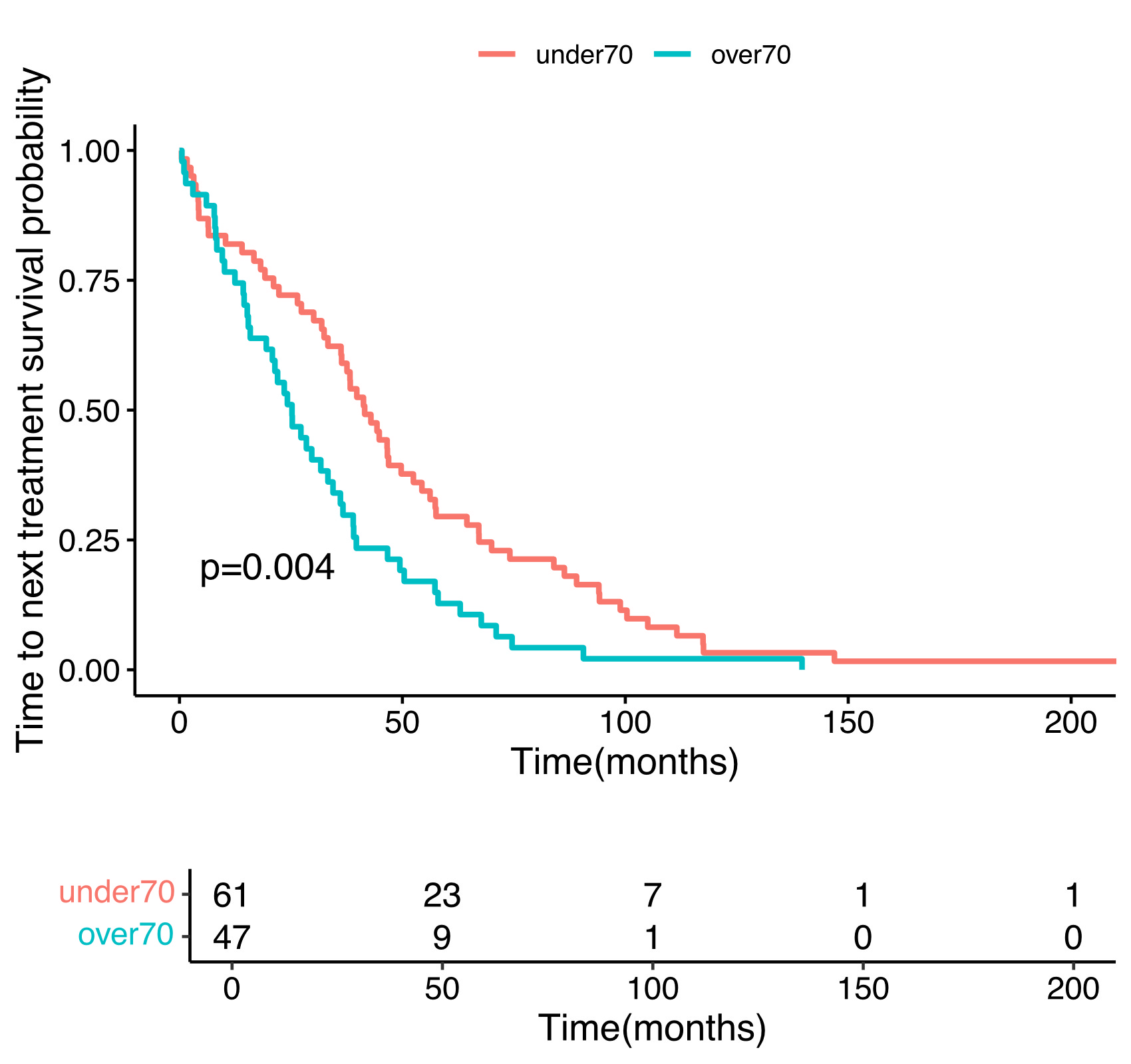
 B)
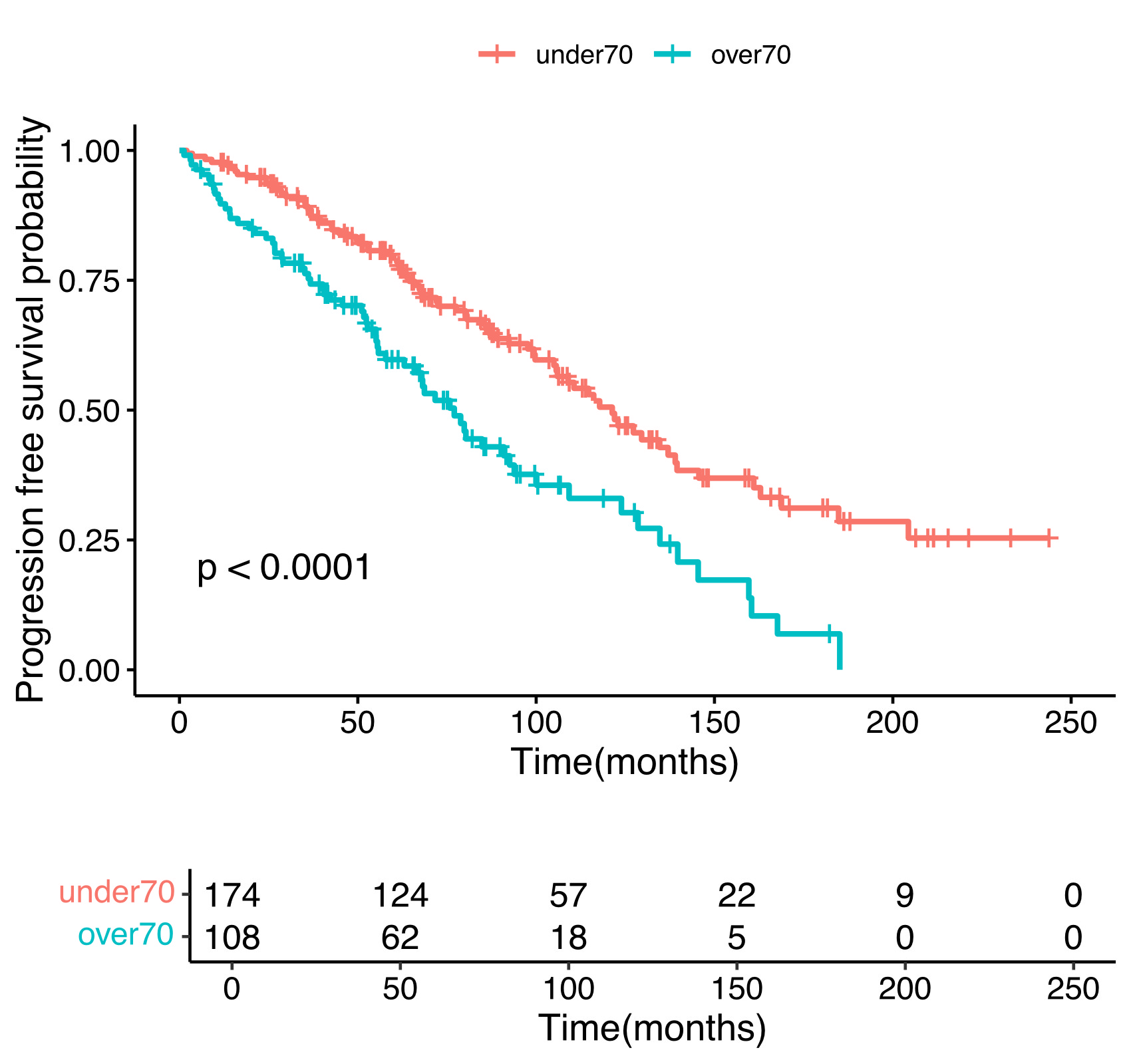
**
2. **
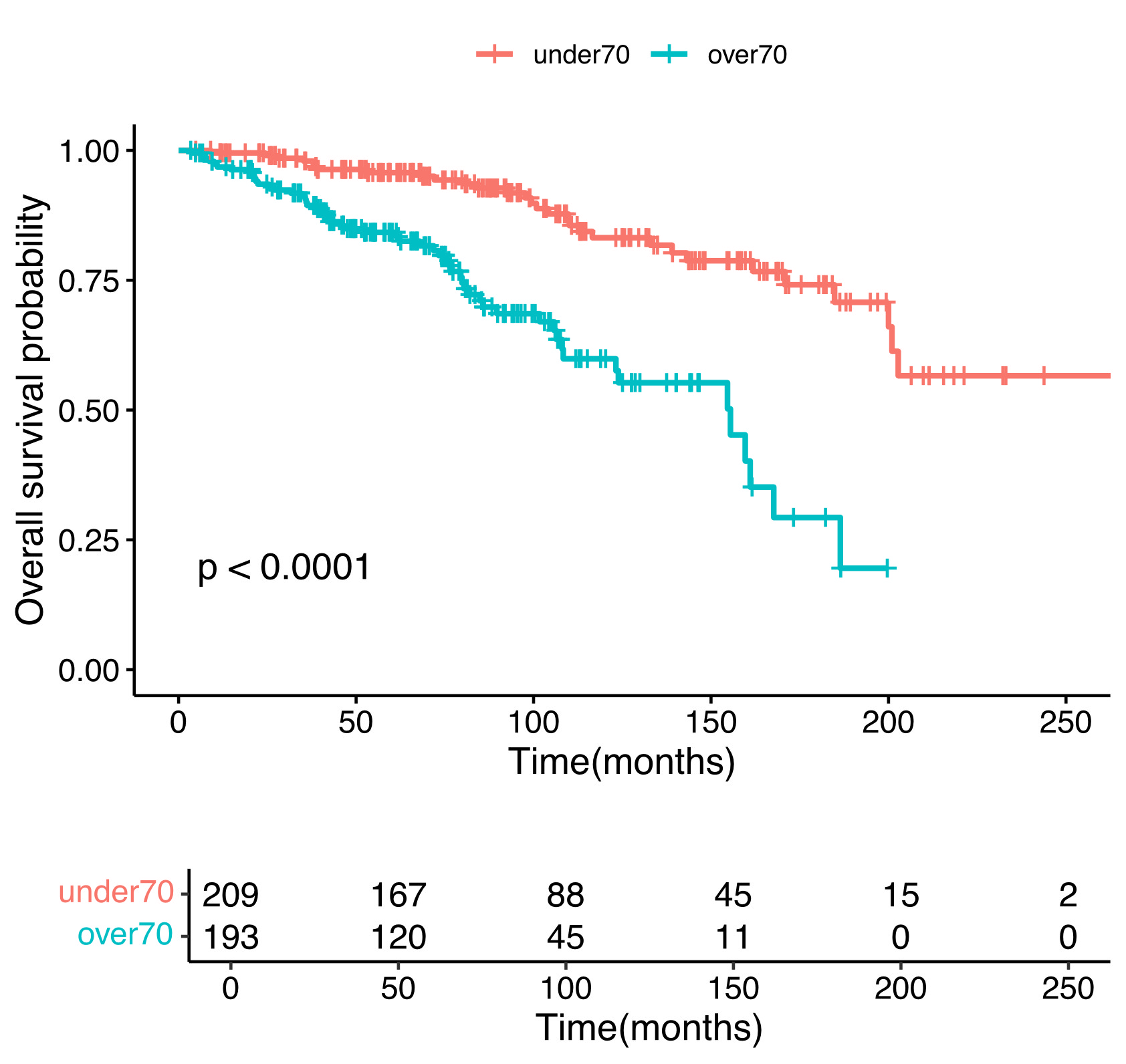
**

**Figure S6.** PFS, OS, TTNT in sWM patients according to the severity of renal dysfunction: mild (blue; eGFR 60-45 ml/min/1.73m^2^), moderate (green; eGFR 45-30 ml/min/1.73m^2^) and severe (red; eGFR < 30 ml/min/1.73m^2^).

1.
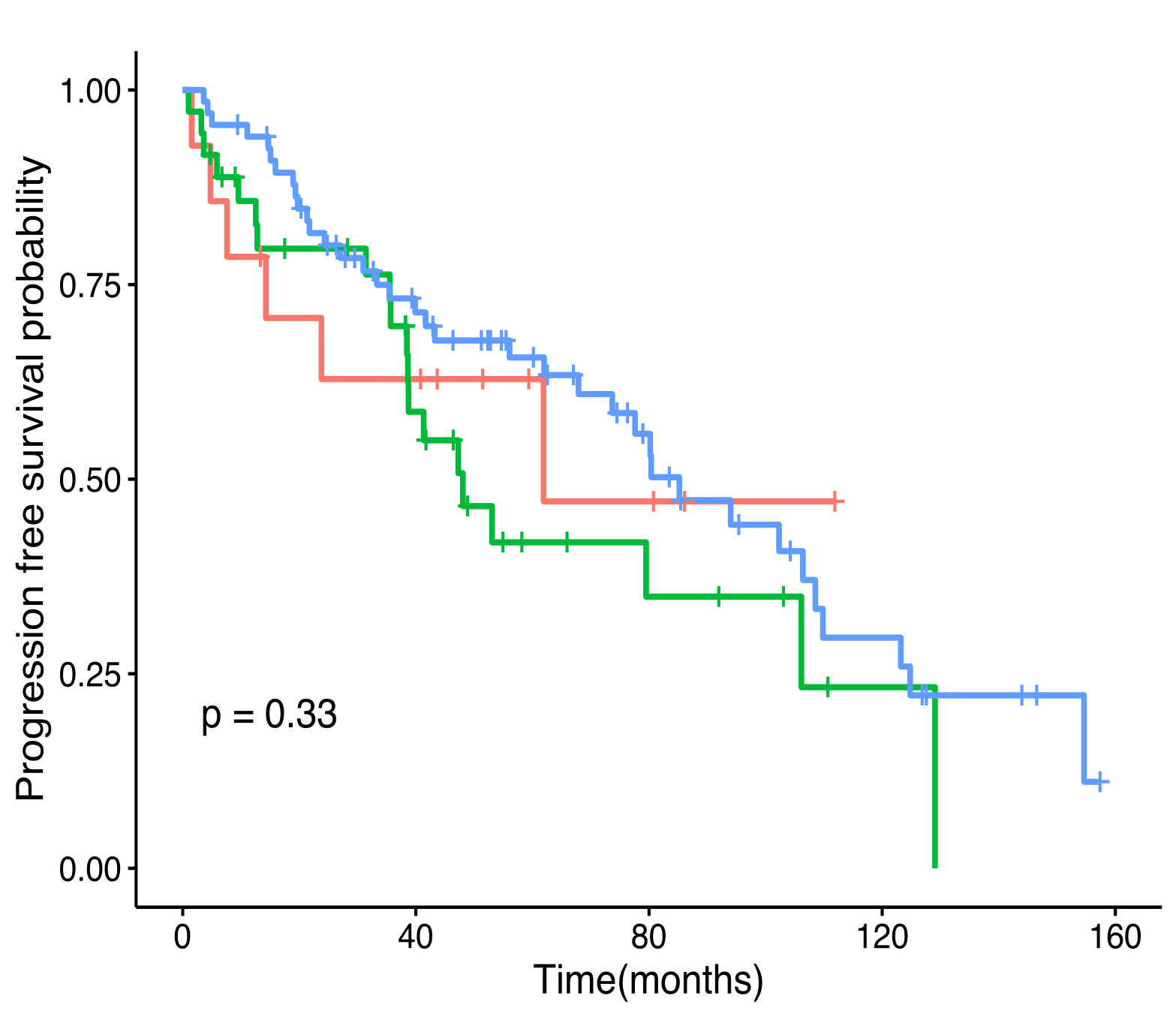
 B)
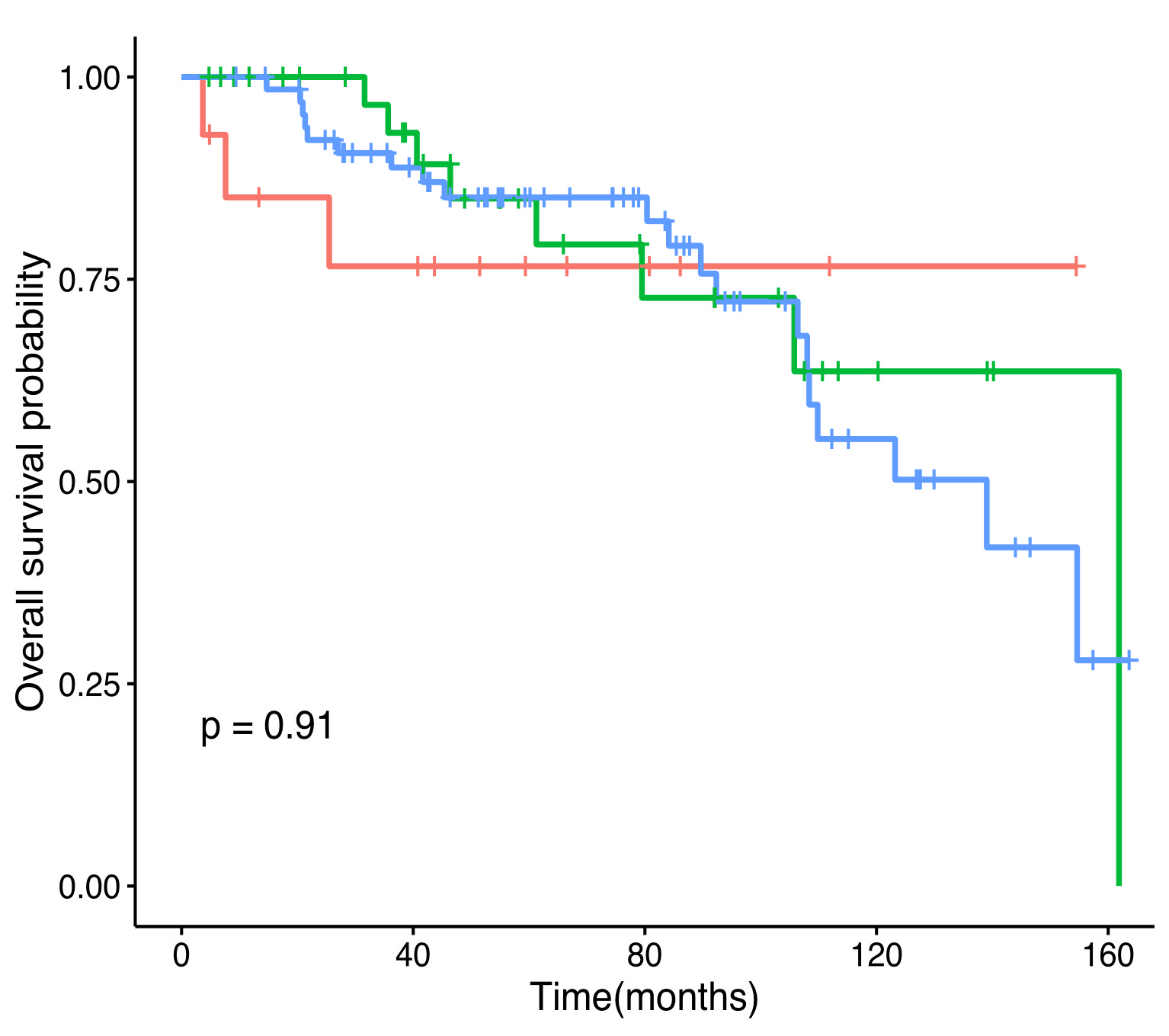

2.
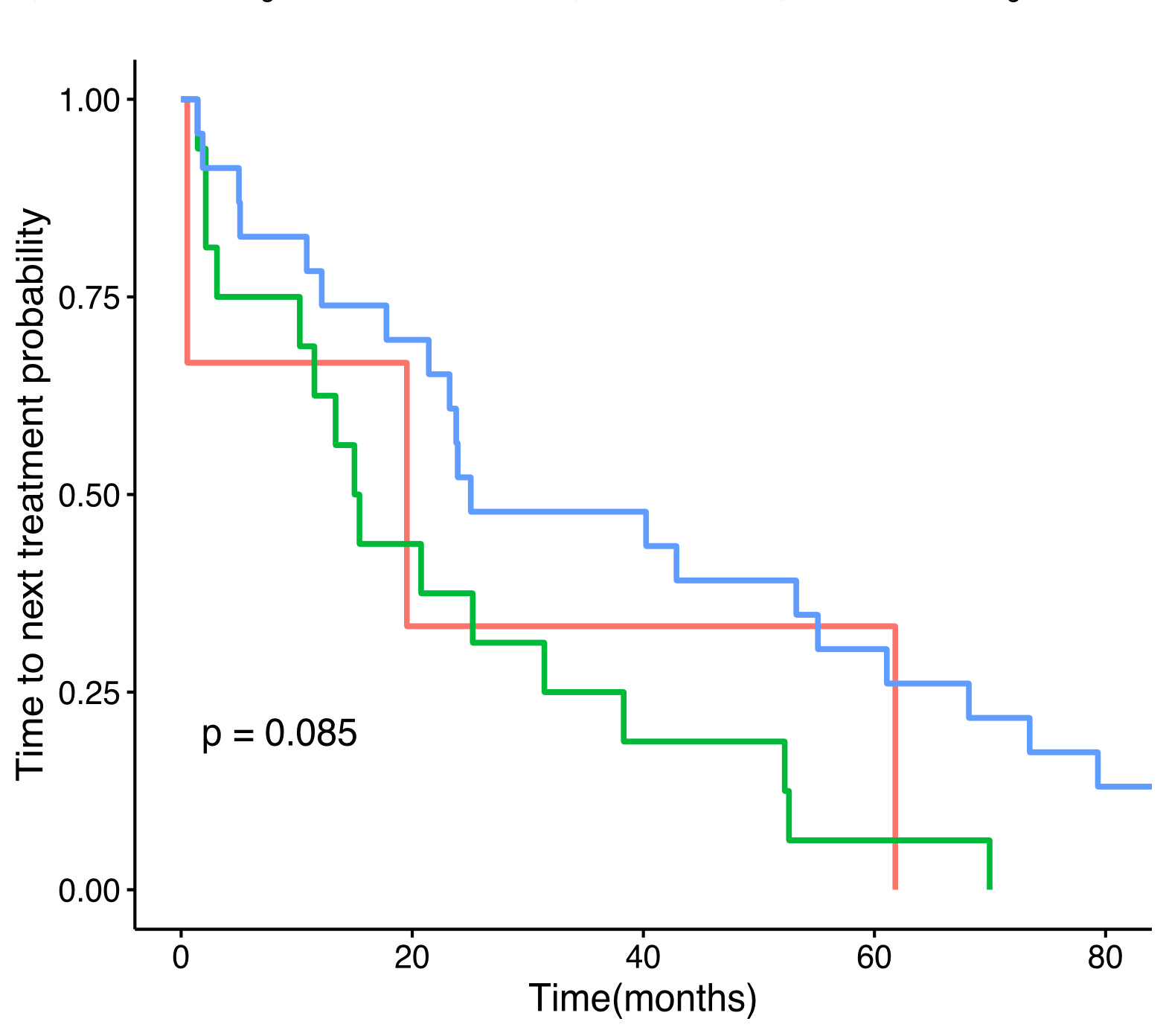


**Figure S7.** TTNT, PFS, OS in sWM cases with (green) and without (red) renal biopsy.

A)
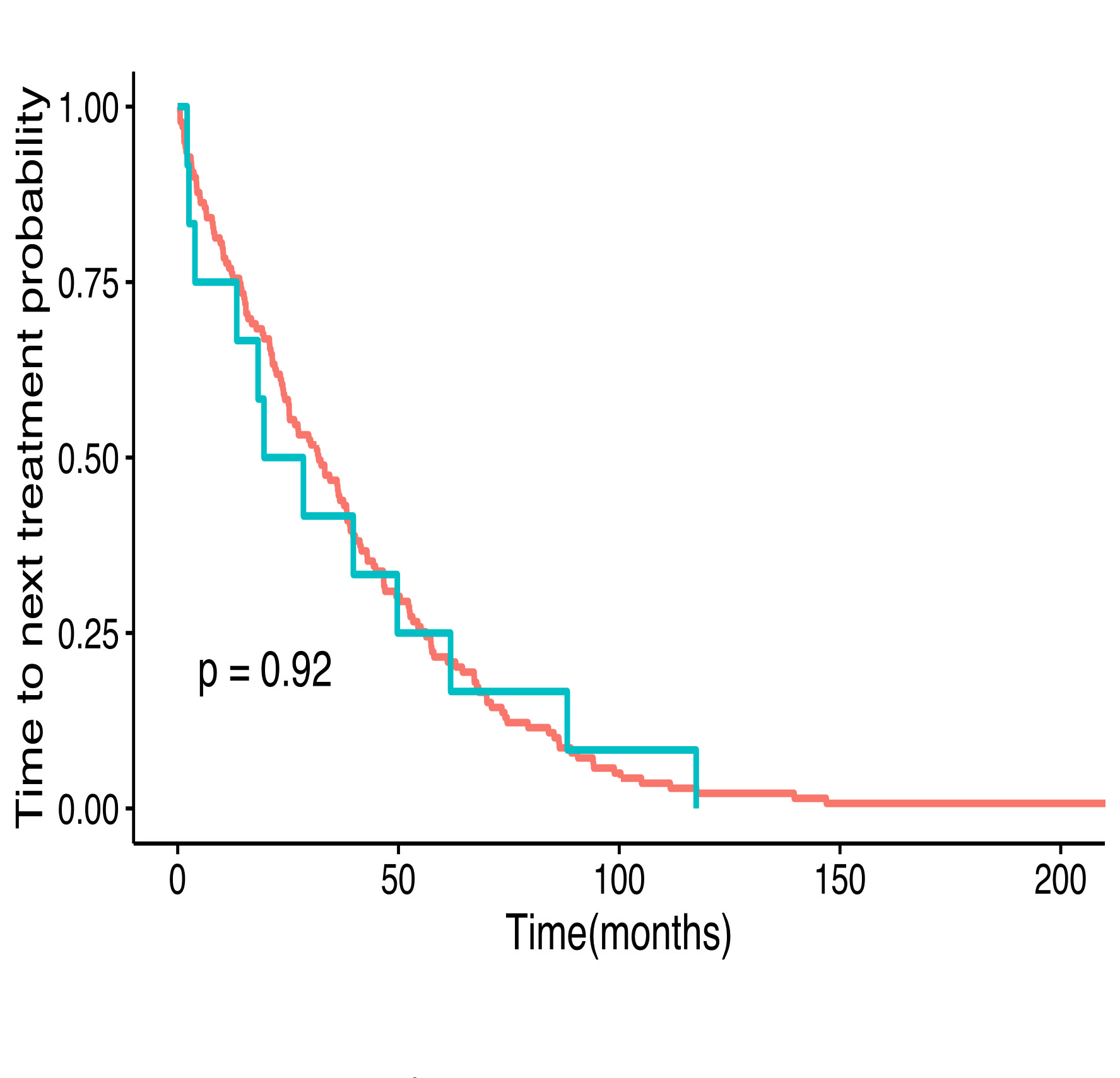
 B)
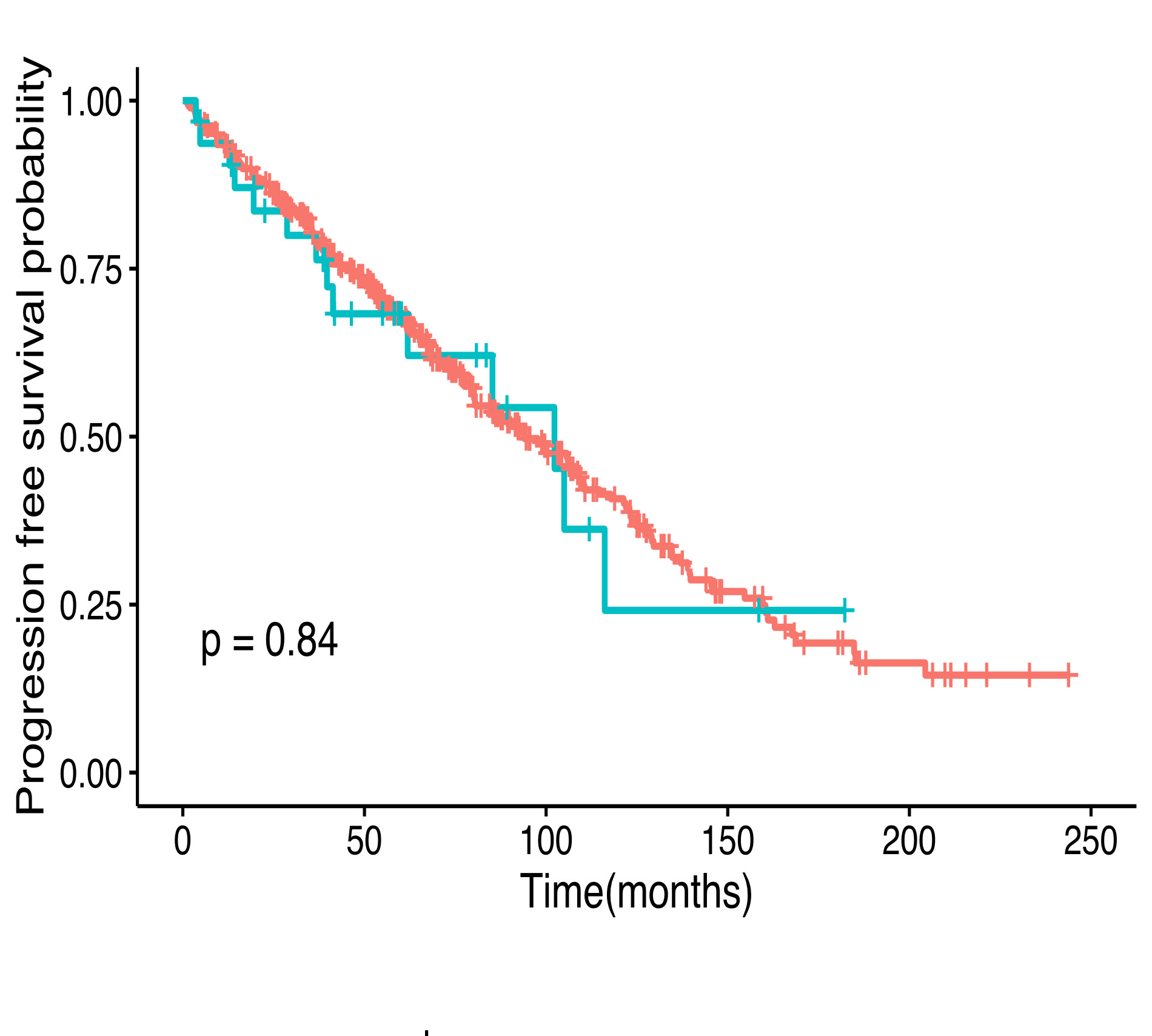


C)
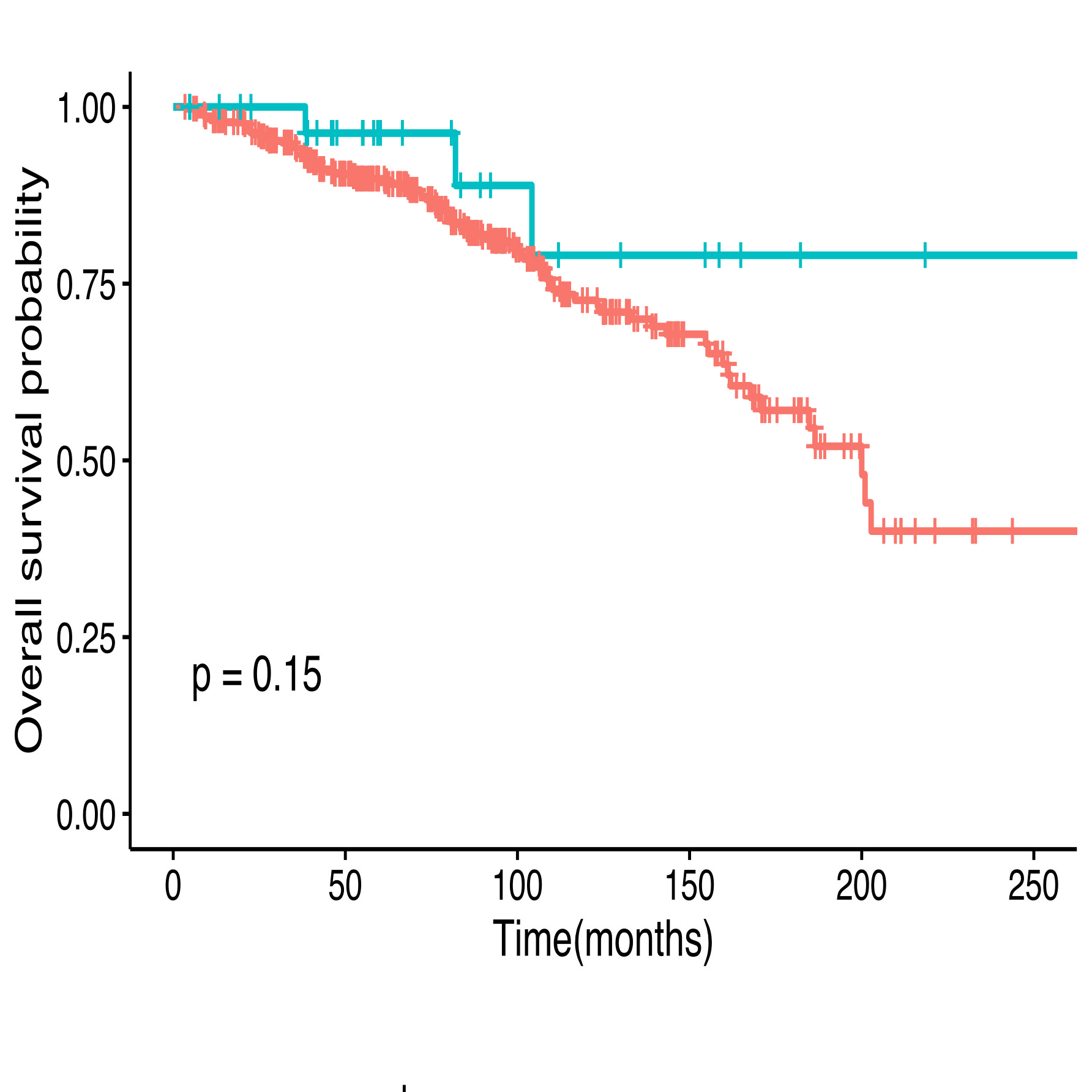


**Figure S8.** Impact of rituximab-bendamustine compared with other regimens on PFS in sWM patients with renal dysfunction subgroup.


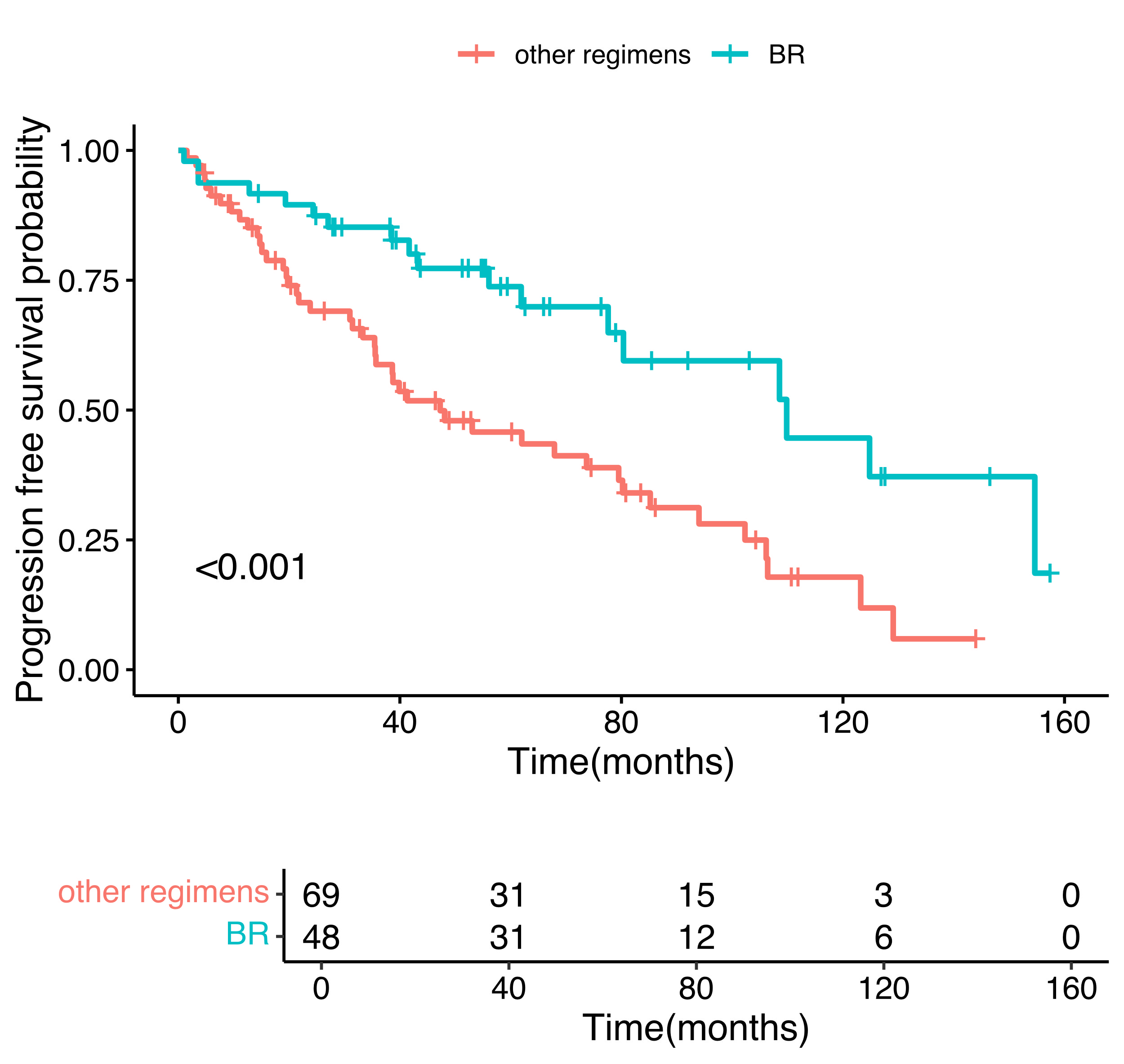


**Table S1. Response rates of BR compared with other regimens in sWM patients with and without renal dysfunction.**

|  | Renal dysfunction | |  | No Renal dysfunction | |  |
| --- | --- | --- | --- | --- | --- | --- |
|  | BR  (48/119) | No BR  (71/119) |  | BR  (126/283) | No BR  (157/283) |  |
| MRR, n (%) | 35/48  (72) | 54/71  (76) | n.s. | 86/126 (68) | 122/157  (78) | 0.07 |
| ORR, n (%) | 40/48 (83) | 58/71 (82) | n.s. | 98/126  (78) | 133/157  (85) | n.s. |
| CR, n (%) | 5/48 | 9/71 | - | 24/118 | 20/152 | - |
| VGPR, n (%) | 5/48 | 6/71 | - | 22/118 | 23/152 | - |
| PR, n (%) | 25/48 | 39/71 | - | 40/118 | 79/152 | - |
| MR, n (%) | 5/48 | 4/71 | - | 12/118 | 11/152 | - |
| SD, n (%) | 8/48 | 7/71 | - | 20/118 | 16/152 | - |
| PD, n (%) | 0/48 | 6/72 | - | 8/118 | 8/152 | - |

BR = bendamustine-rituximab, MRR = major response rate, ORR = overall response rate, CR = complete response, VGPR = very good partial response, PR = partial response, MR = minor response, SD = stable disease, PD = progressive disease

**Table S2. Response rates of chemioimmunotherapy (CIT) compared with other regimens in sWM patients with and without renal dysfunction.**

|  | Renal dysfunction | |  | No renal dysfunction | |  |
| --- | --- | --- | --- | --- | --- | --- |
|  | CIT  (75/119) | No CIT  (44/119) |  | CIT  (191/283) | No CIT  (92/283) |  |
| MRR, n (%) | 57/75  (76) | 28/44  (64) | n.s. | 135/191  (71) | 73/92 (79) | 0.001 |
| ORR, n (%) | 67/75 (89) | 31/44 (70) | 0.009 | 153/191 (80) | 78/92 (85) | n.s. |
| CR, n (%) | 10/75 | 4/41 | - | 34/191 | 10/92 | - |
| VGPR, n (%) | 7/75 | 3/41 | - | 33/191 | 12/92 | - |
| PR, n (%) | 43/75 | 21/41 | - | 68/191 | 51/92 | - |
| MR, n (%) | 7/75 | 3/41 | - | 18/191 | 5/92 | - |
| SD, n (%) | 7/75 | 7/41 | - | 25/191 | 8/92 | - |
| PD, n (%) | 1/75 | 6/41 | - | 13/191 | 6/92 | - |

CIT = chemoimmunotherapy, MRR = major response rate, ORR = overall response rate, CR = complete response, VGPR = very good partial response, PR = partial response, MR = minor response, SD = stable disease, PD = progressive disease

**Table S3. First line therapy regimens, dose and cycle modifications, and response rates in sWM patients with renal dysfunction stratified by renal biopsy status.**

|  | **Biopsed**    n = 21 | **Not biopsed**  n = 98 | P value |
| --- | --- | --- | --- |
| CIT, n (%) | 15/21 (68) | 59/98 (60) | n.s. |
| BR, n (%) | 7/21 (33) | 41/98 (42) | n.s. |
| RCD, n (%) | 8/21 (38) | 18/98 (18) | **0.05** |
| R-Chlorambucil, n (%) | 0/21 (0) | 2/98 (2) | n.s. |
| Bortezomib-based regimens*, n (%) | 4/21 (19) | 1/98 (1) | **< 0.001** |
| Other**, n (%) | 1/21 (5) | 34/98 (35) | n.s. |
|  |  |  |  |
| Treatment modification, n (%) | 7/21 (30) | 26/98 (27) | n.s. |
| Dose reduction, n (%) | 5/21 (24) | 15/98 (15) | n.s. |
| Cycle reduction, n (%) | 4/21 (19) | 17/98 (17) | n.s. |
| Retreatment, n (%) | 8/21 (38) | 35/98 (36) | n.s. |
|  |  |  |  |
| MRR, n (%) | 17/21 (81) | 74/99 (75) | n.s. |
| ORR, n (%) | 19/21 (90) | 84/99 (85) | n.s. |
| CR, n (%) | 5/21 (24) | 10/98 (10) | - |
| VGPR, n (%) | 2/21 (9) | 8/98 (8) | - |
| PR, n (%) | 10/21 (48) | 54/99 (55) | - |
| MR, n (%) | 2/21 (10) | 10/98 (10) | - |
| SD, n (%) | 0/21 (0) | 10/98 (10) | - |
| PD, n (%) | 2/21 (10) | 6/98 (6) | - |

CIT = chemoimmunotherapy, BR = bendamustine-rituximab, RCD = Rituximab-Cyclophoshamide-Dexamethasone, MRR = major response rate, ORR = overall response rate, CR = complete response, VGPR = very good partial response, PR = partial response, MR = minor response, SD = stable disease, PD = progressive disease

**Table S4. Median Age of sWM patients according to renal dysfunction severity.**

| **Renal Dysfunction** | **Median Age (IQR)** |
| --- | --- |
|  |  |
| Low (ClCr 60-45 ml/min/1.73m^2^) | 75.3 (67.9-79.5) |
| Intermediate (ClCr 45-30 ml/min/1.73m^2^) | 78.5 (71.4-80.9) |
| Severe (ClCr < 30 ml/min/1.73m^2^) | 78.5 (71.8-81.2) |

ClCr = Creatinine Clearance ; WM = Waldenström Macroglobulinemia ; IQR = Interquartile range

**Table S5. Histological and clinical characteristics of renal-biopsied sWM cases.**

|  | ***Histology*** | ***Time from WM diagnosis***  ***(months)*** | ***Creatinine (mg/dL)*** | ***eGFR (ml/min)*** | ***Proteinuria (mg/24h)*** | ***Therapy*** | ***Hematological***  ***response*** | ***Renal***  ***response*** |
| --- | --- | --- | --- | --- | --- | --- | --- | --- |
| ***#1*** | ***Tubulointerstitial infiltration*** | ***37,00*** | ***1,13*** | ***80*** | ***1000*** | ***DRC*** | ***PR?*** | ***Stable*** |
| ***#2*** | ***Non cryoglobulinemic GN*** | ***1,00*** | ***2,56*** | ***47*** | ***3370*** | ***DRC*** | ***PD*** | ***Stable*** |
| ***#3*** | ***Tubulointerstitial infiltration*** | ***21,00*** | ***1,20*** | ***88*** | ***N/A*** | ***BRD*** | ***PR*** | ***Stable*** |
| ***#4*** | ***Tubulointerstitial infiltration*** | ***0,50*** | ***4,46*** | ***17*** | ***N/A*** | ***BR*** | ***SD*** | ***Stable*** |
| ***#5*** | ***Non cryoglobulinemic GN*** | ***2,00*** | ***1,03*** | ***66*** | ***190*** | ***BR*** | ***SD*** | ***Stable*** |
| ***#6*** | ***Non cryoglobulinemic GN*** | ***120,00*** | ***5,59*** | ***9*** | ***N/A*** | ***BR*** | ***VGPR*** | ***Stable*** |
| ***#7*** | ***Tubulointerstitial infiltration*** | ***0,25*** | ***1,60*** | ***45*** | ***N/A*** | ***BR*** | ***CR*** | ***N/A*** |
| ***#8*** | ***Tubulointerstitial infiltration*** | ***20,00*** | ***0,90*** | ***110*** | ***8600*** | ***BRD*** | ***PD*** | ***Stable*** |
| ***#9*** | ***AL amyloidosis*** | ***19,00*** | ***0,63*** | ***60*** | ***4500*** | ***DRC*** | ***CR*** | ***Stable*** |
| ***#10*** | ***AL amyloidosis*** | ***0,25*** | ***0,94*** | ***102*** | ***200*** | ***BRD*** | ***PR*** | ***Stable*** |
| ***#11*** | ***Non cryoglobulinemic GN + tubulointerstitial infiltration*** | ***60,00*** | ***2,60*** | ***25*** | ***N/A*** | ***DRC*** | ***PR*** | ***Worse*** |
| ***#12*** | ***Non cryoglobulinemic GN*** | ***0,25*** | ***3,50*** | ***10*** | ***1000*** | ***BDR*** | ***PR*** | ***Worse*** |
| ***#13*** | ***Non cryoglobulinemic GN + LCDD*** | ***2,00*** | ***2,50*** | ***19*** | ***70*** | ***DRC*** | ***PR*** | ***Better*** |
| ***#14*** | ***Non cryoglobulinemic GN*** | ***0,25*** | ***1,60*** | ***48*** | ***3000*** | ***DRC*** | ***SD*** | ***Stable*** |
| ***#15*** | ***Cryoglobulinemic GN*** | ***0,25*** | ***1,50*** | ***45*** | ***480*** | ***DRC*** | ***PR*** | ***Stable*** |
| ***#16*** | ***Tubulointerstitial infiltration*** | ***13,00*** | ***1,90*** | ***42*** | ***1390*** | ***DRC*** | ***PR*** | ***Better*** |
| ***#17*** | ***PGNMID*** | ***18,00*** | ***0,90*** | ***81*** | ***2620*** | ***BRD*** | ***PR*** | ***Better*** |
| ***#18*** | ***AL amyloidosis*** | ***0,25*** | ***6,00*** | ***7*** | ***N/A*** | ***BRD*** | ***PD*** | ***Worse*** |
| ***#19*** | ***AL amyloidosis*** | ***0,25*** | ***1,60*** | ***35*** | ***3500*** | ***BR*** | ***PR*** | ***Worse*** |
| ***#20*** | ***Nephroangiosclerosis*** | ***96,00*** | ***1,27*** | ***63*** | ***980*** | ***DRC*** | ***SD*** | ***Stable*** |
| ***#21*** | ***AL amyloidosis*** | ***0,25*** | ***2,76*** | ***27*** | ***8390*** | ***BDR*** | ***PD*** | ***Worse*** |
| ***#22*** | ***AL amyloidosis*** | ***0,25*** | ***1,90*** | ***22*** | ***7740*** | ***DRC*** | ***PR*** | ***Worse*** |
| ***#23*** | ***Non cryoglobulinemic GN*** | ***17,00*** | ***2,40*** | ***29*** | ***270*** | ***BR*** | ***VGPR*** | ***Better*** |
| ***#24*** | ***Tubulointerstitial infiltration*** | ***0,25*** | ***0,55*** | ***99*** | ***N/A*** | ***BR*** | ***PR*** | ***Stable*** |
| ***#25*** | ***AL amyloidosis*** | ***1,00*** | ***3,00*** | ***40*** | ***3000*** | ***BR*** | ***PR*** | ***Better*** |
| ***#26*** | ***Tubulointerstitial infiltration*** | ***0,25*** | ***1,49*** | ***30*** | ***1360*** | ***DRC*** | ***PR*** | ***Stable*** |
| ***#27*** | ***AL amyloidosis*** | ***96,00*** | ***1,60*** | ***53*** | ***500*** | ***BR*** | ***PR*** | ***Stable*** |
| ***#28*** | ***Non cryoglobulinemic GN*** | ***1,00*** | ***1,91*** | ***38*** | ***340*** | ***BR*** | ***SD*** | ***Stable*** |
| ***#29*** | ***Tubulointerstitial infiltration*** | ***108,00*** | ***1,66*** | ***40*** | ***2030*** | ***BDR*** | ***PR*** | ***Stable*** |
| ***#30*** | ***Non cryoglobulinemic GN + LCDD*** | ***1,00*** | ***4,57*** | ***11*** | ***16680*** | ***BDR*** | ***PR*** | ***Better*** |
| ***#31*** | ***AL amyloidosis*** | ***12,00*** | ***1,09*** | ***80*** | ***180*** | ***BR*** | ***PR*** | ***Stable*** |
| ***#32*** | ***AL amyloidosis*** | ***108,00*** | ***0,98*** | ***85*** | ***130*** | ***Leukeran-R*** | ***PR*** | ***Stable*** |
| ***#33*** | ***Tubulointerstitial infiltration*** | ***1,00*** | ***0,68*** | ***90*** | ***16500*** | ***DRC*** | ***VGPR*** | ***Stable*** |

GN = glomerulonephritis; LCDD = light chain deposition disease; PGNMID = Proliferative Glomerulonephritis with Monoclonal Immunoglobulin Deposits; BR = bendamustine, rituximab; BDR = bortezomib, dexamethasone, rituximab; PR = partial response; SD = stable disease; CR = complete response; VGPR = very good partial response; PD = progressive disease; Worse = 25% of eGFR decreasing after 1^st^ line therapy ; Stable = eGFR stabilizing between +25% and – 25% after 1^st^ line therapy; Better = 25% of eGFR increasing after 1^st^ line therapy.
